# Strategy to Find Molecular Signatures in a Small Series of Rare Cancers: Validation for Radiation-Induced Breast and Thyroid Tumors

**DOI:** 10.1371/journal.pone.0023581

**Published:** 2011-08-11

**Authors:** Nicolas Ugolin, Catherine Ory, Emilie Lefevre, Nora Benhabiles, Paul Hofman, Martin Schlumberger, Sylvie Chevillard

**Affiliations:** 1 CEA, DSV, IRCM, SREIT, Laboratoire de Cancérologie Expérimentale, BP6, Fontenay-aux-Roses, France; 2 INSERM ERI-21, Nice, France; 3 University of Nice-Sophia Antipolis, IFR 50, Nice, France; 4 Laboratory of Clinical and Experimental Pathology and CHU-CRLCC-UNSA Tumour/Tissue Bank of Nice Area, Louis Pasteur Hospital, Nice, France; 5 Department of Nuclear Medicine and Endocrine Oncology, Institut Gustave Roussy, Villejuif, France; University of Georgia, United States of America

## Abstract

Methods of classification using transcriptome analysis for case-by-case tumor diagnosis could be limited by tumor heterogeneity and masked information in the gene expression profiles, especially as the number of tumors is small. We propose a new strategy, EMts_2PCA, based on: 1) The identification of a gene expression signature with a great potential for discriminating subgroups of tumors (EMts stage), which includes: a) a learning step, based on an expectation-maximization (EM) algorithm, to select sets of candidate genes whose expressions discriminate two subgroups, b) a training step to select from the sets of candidate genes those with the highest potential to classify training tumors, c) the compilation of genes selected during the training step, and standardization of their levels of expression to finalize the signature. 2) The predictive classification of independent prospective tumors, according to the two subgroups of interest, by the definition of a validation space based on a two-step principal component analysis (2PCA). The present method was evaluated by classifying three series of tumors and its robustness, in terms of tumor clustering and prediction, was further compared with that of three classification methods (Gene expression bar code, Top-scoring pair(s) and a PCA-based method). Results showed that EMts_2PCA was very efficient in tumor classification and prediction, with scores always better that those obtained by the most common methods of tumor clustering. Specifically, EMts_2PCA permitted identification of highly discriminating molecular signatures to differentiate post-Chernobyl thyroid or post-radiotherapy breast tumors from their sporadic counterparts that were previously unsuccessfully classified or classified with errors.

## Introduction

In oncology, tumor classification is key when assessing prognosis, defining the most appropriate treatment, identifying sensitive and resistant patients or comparing treatments [1]–[3]. Currently, histological criteria for tumor sections and fine-needle biopsy specimens do not always greatly improve tumor classification. Numerous studies have sought to enrich these criteria with data from molecular biology, comparative genomic hybridization, and transcriptomic and/or proteomic analysis. In particular, tumors have been successfully studied and classified using DNA chip analysis. However, the routine use of gene expression data to classify tumors is limited by the background noise inherent in the technique [4], by the fact that gene expression varies within a given subgroup of tumors, and because most levels of gene expression do not differ significantly from one group to another. Consequently, these difficulties increase the two challenges when using microarrays in tumor classification, namely: 1) identification of the gene signature to discriminate two subgroups of tumors, 2) objective validation of the signature, diagnosing independent tumors [5]. It is worth mentioning that most of the commonly used methods select genes expressed differentially between two subgroups, unless the intra-group heterogeneity is not high enough [6]–[9]. To circumvent these limitations, some methods use permutations to minimize the effect of the heterogeneity between the tumors [10]. However, this could be problematical in two cases: if the genes included in the signature vary substantially depending on the different permutations, since divergence may lead to lack of a common signature, and if large tumor overlap, among the permutations, results in biased selection of genes. These problems could lead to the impossibility of classifying independent tumors for the validation, notably with the usually applied methods such as hierarchical clustering and PCA analysis. Moreover, it may also occur that the information of interest is masked in the gene expression profiles [5], [11], [12]. All these difficulties are enhanced by working with small series, which is necessarily the case for rare diseases.

The EMts_2PCA method, presented hereafter, was specially designed to overcome these limitations. It was applied to two subgroups of human thyroid tumors (follicular thyroid adenoma (FTA) and papillary thyroid carcinoma (PTC)), to define a biologically relevant gene signature and to blindly classify a testing set of independent tumors. Moreover, the accuracy of the EMts_2PCA method was also tested by the classification of the tumors of two already published series. The first series of post-Chernobyl and sporadic PTC was either not classified using the usual methods of unsupervised or supervised tumor classification [11], or classified with errors using methods such as generalized partial least-square (GPLS), random forest (RF), linear kernel support vector machine (LKSVM), prediction analysis of microarray (PAM) [12], and the second series of post-radiotherapy breast tumors was classified with errors using an unsupervised hierarchical clustering and subsequent supervised classification (SAM) [13]. In addition, analyzing the same three series of tumors, we have compared the performance of the EMts_2PCA method with three alternative methods, gene expression bar code [14], top-scoring pair (TSP) [15] and a PCA-based method [16]. Advantages and weaknesses of these methods are discussed, but in any case our method performed best in analysis of a small series of samples.

## Results

The details of the process are given for the follicular thyroid adenoma and papillary thyroid carcinoma (FTA/PTC) series. For other sample series, only signature and scoring will be given.

### EMts_2PCA analysis: Identification of a gene expression signature with a great potential for discriminating subgroups of tumors (EMts stage)

#### Learning step to screen for candidate genes

The 54 tumors analyzed in this study were divided into two sets: a learning/training set of known histology subgroups, comprising 13 follicular thyroid adenomas (FTAs) and 13 papillary thyroid carcinomas (PTCs), and a testing set of 28 independent tumors. After microarray hybridization, the hybridization signals were acquired and normalized, and the calculated expression ratios were arranged in expression matrices [8]. From the learning/training set, 169 combinatorial matrices of 10 tumors (5 FTAs versus 5 PTCs = 10-tumor matrix) were built as described in (Material and Methods: Learning step/Search for set of candidate genes). For each 10-tumor matrix, a weakly discriminating filter, based on a t-test with permutations, retained the genes expressed differentially from those belonging to the noise [17].

The filtering t-test selected between 1604 and 2800 genes depending on the combinatorial matrices. Then, for each of the 169 filtering 10-tumor matrices, an EM algorithm was applied to select the genes which were always expressed in the same way (induced or down-regulated) within the PTC subgroup and in an opposite way (down-regulated or induced, respectively) in the FTA subgroup. Then, the 169 10-tumor matrices were reduced to 169 training-matrices of the selected genes. The training matrices used in the training step comprised on average 331 candidate genes (standard deviation 290).

#### Training step

The next step, the training step, determined which training matrices have the higher potential to classify tumors (Material and Methods: Training step with internal cross-validation). This training step consisted in using each training matrix to classify, by a two-step PCA stage (2PCA), the tumors of the learning set that did not belong to the considered matrix (training tumors) (Material and Methods: Classification of training tumors). Each training tumor of a given training matrix and all the tumors of this training matrix were projected into the training space. The training tumor was then automatically attributed to either the FTA or the PTC subgroup, or assigned to neither the FTA nor the PTC subgroup, as a function of the RMS values (Material and Methods: Classification rules as a function of the RMS). The training matrix was validated when at least one training tumor was correctly classified and none was misclassified (the classification of the other tumors must be unattributed). Applying this rule, 121 of 169 training matrices were validated and used for the search for the final signature.

#### Gene compilation and standardization for the final signature

The search for the unique set of genes (final signature) for the tumor classification needed a standardization step to smooth the gene expression heterogeneity. So, the standardized matrix ξ was calculated from the compilation of the 121 validated training matrices, each element (i,j) of the matrix ξ being the mean of the equivalent elements (i_h_,j_h_) of all training matrices, as described in Material and Methods (Standardization). After the standardization, the single signature was composed of the genes of the matrix ξ with the greater frequencies of relevance F(i). The greater frequencies of relevance are defined by the asymptote to 1 of the cumulative distribution function of the F(i,j) matrix, and by the limit (mF+2vF) for which the function becomes asymptotic. Above this limit, the observed F(i,j) become statistically different and higher as compared with the mean frequency mF. In the present analysis, this point corresponded to p = 0.001, and 227 genes (230 probes) were selected by this threshold and constitute the final signature (Table S1).

### EMts_2PCA analysis: Validation by the predictive classification of testing tumors by 2sPCA

**Figure 1:**
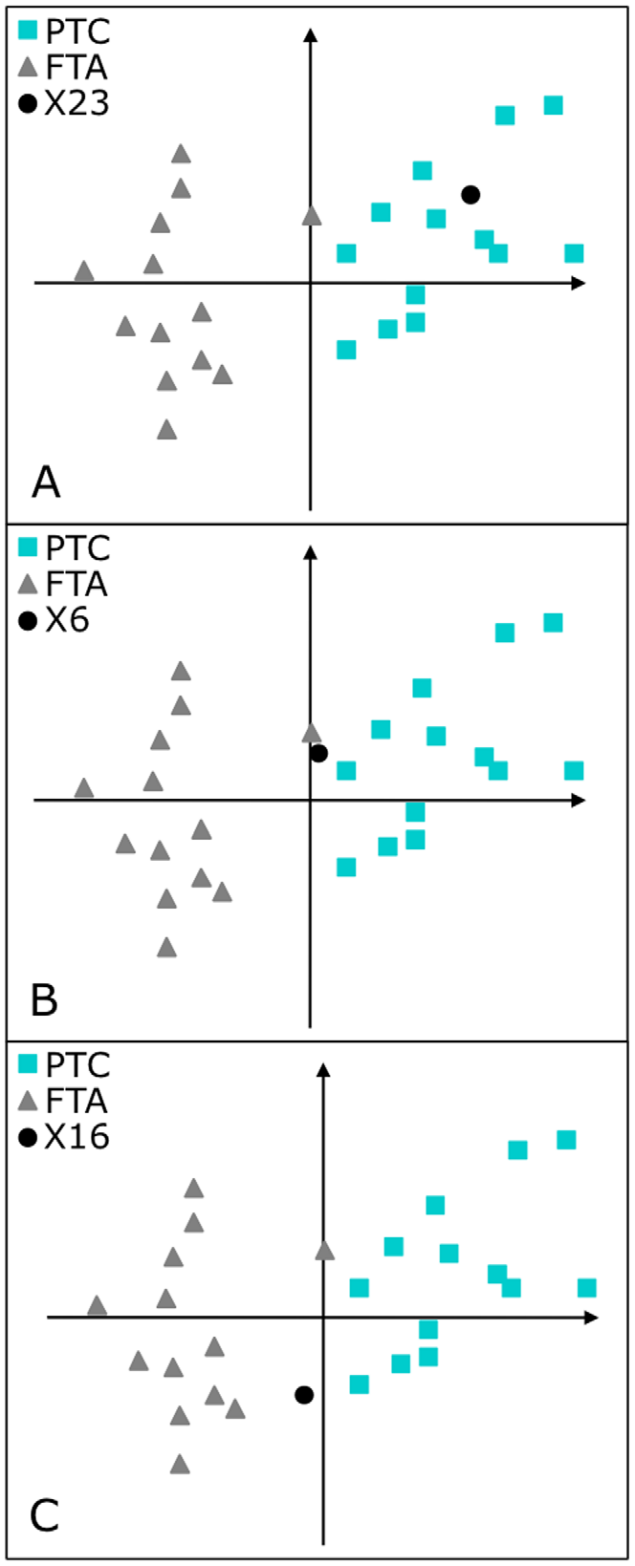
Two-step PCA positioning of the testing tumors: follicular thyroid adenoma and papillary thyroid carcinoma. Examples of positioning of tumors ×23, ×6 and ×16 (dot) in the validation space. The FTA (triangle) populated area and the distinct PTC (square) populated area delimited the spatial distribution of the learning tumors in the validation space (Material and Methods 4.3.1b). **A**: one example of a correctly classified tumor (×23). **B**, **C**: outlier tumors (×6, ×16) positioned in the validation space between the two subgroups.

**Figure 2:**
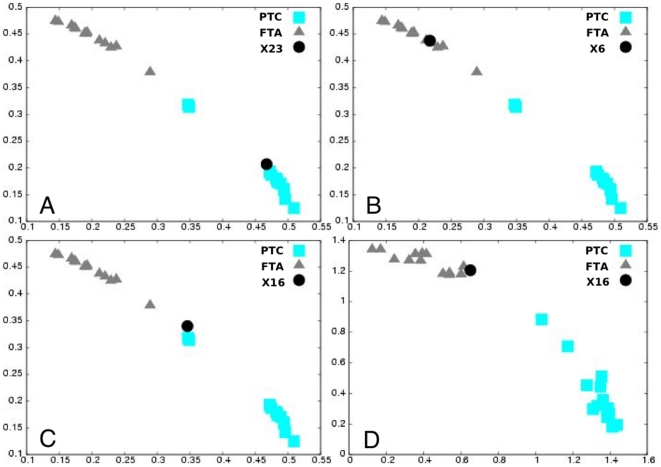
RMS classification of the testing tumors: follicular thyroid adenoma and papillary thyroid carcinoma. Testing tumor (×23, ×6 and ×16) (dot) classification considering all eigenvectors (validation space) in the RMS computation (**A**, **B** and **C**), or considering the combination of eigenvectors that maximized the asymmetry between the two groups and minimized the heterogeneity within each subgroup together (new validation space) (**D**). Scatter plot of RMS_FTA_^matrix^ as a function of the RMS_PTC_^matrix^ for the learning tumors and the RMS_FTA_^class^ as a function of the RMS_PTC_^class^ of the considered testing tumor. The RMS values of the training tumors fall into two distinct groups (FTA, triangle and PTC, square). **A**: good classification of the ×23 tumor. **B**: good classification of the ×6 tumor without considering RMS (Figure 1B). **C**: outlier classification of the ×16 tumor in the validation space but **D**: good classification of the ×16 tumor considering the new validation space.

A matrix Ψ' of learning tumors was built from the standardized matrix ξ, restricted to the genes of the final signature, and projected into the validation space (Material and Methods: Classification of testing tumors). The validation space clearly separated the tumors of the learning set into two subgroups (FTA and PTC) (Figure 1). Each tumor of the testing set to be classified in a blinded evaluation was then projected into the validation space, already containing the tumors of the learning set. Of the 28 tumors to be classified, 26 were unambiguously located in their FTA or PTC subgroup. Two outlier tumors were positioned in the validation space between the two subgroups (tumors ×6 and ×16, Figure 1B, 1C) (Material and Methods: Aid to classification of testing tumors). By considering all eigenvectors in the RMS computation (Material and Methods: Classification rules as a function of the RMS), one of these remaining tumors (tumor ×6), was automatically attributed to the FTA subgroup (Figure 2B), while the last tumor (tumor ×16), could not be attributed (Figure 2C). However, by selecting the combination of eigenvectors that maximized the asymmetry between the two groups and minimized the heterogeneity within each subgroup (Material and Methods: Selection of eigenvectors to improve the classification), the new validation space and the RMS computation from these vectors (Figure 2D) clearly classified the ×16 tumor in the FTA subgroup without modifying the classification of any other tumors.

In conclusion, we found a 227-gene signature by analyzing a series of 26 tumors which was further validated for an independent series of 28 tumors. The EMts_2PCA method is robust since after the histology code-break by the clinicians all 28 tumors were found to be correctly predicted (Table S2A).

To demonstrate the efficiency of our method, we analyzed two previously analyzed and published datasets, a sporadic or post-Chernobyl PTC series of tumors (GSE3950, [12]) and a post-radiotherapy series of breast cancers (E-NCMF-30, [13]).

### EMts_2PCA analysis of post-Chernobyl PTC series

We chose the dataset of 26 sporadic or post-Chernobyl PTCs because the authors could not find a discriminating signature using several standard bioinformatic methods of supervised and unsupervised classification [11], [12]. However, by reading the manuscript, the existence of a signature could be suspected since, by applying an empirical signature elaborated from previously published stress-specific signatures on lymphocytes, these 26 samples were roughly classified by the authors [12], [18].

**Figure 3:**
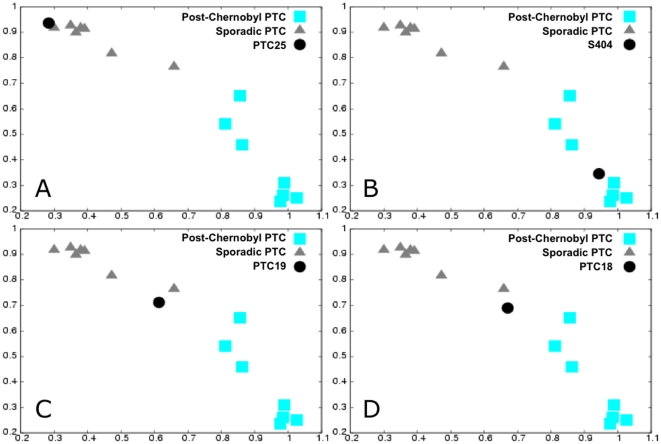
RMS classification of the testing tumors: sporadic and post-Chernobyl papillary thyroid carcinoma. Testing tumors (sporadic PTCs, PTC18, PTC19 and PTC25 and post-Chernobyl PTC, S404) (dot) classification considering all eigenvectors (validation space) in the RMS computation. Scatter plot of RMS_sporadic_^matrix^ as a function of the RMS_chernobyl_^matrix^ for the learning tumors and the RMS_sporadic_^class^ as a function of the RMS_chernobyl_^class^ of the considered testing tumor. The RMS values of the training tumors fall into two distinct groups (sporadic PTC, triangle and post-Chernobyl PTC, square). **A**, **B**, **C**: examples of correctly classified tumors, PTC25, S404 and PTC19, respectively. **D**: outlier classification of the PTC18 tumor.

A signature of 106 genes (109 probes) was found by applying the EMts stage to a learning/training set of 13 tumors (Table S3). The robustness of this signature was assessed by the case-by-case 2PCA classification of an independent testing set composed of the 13 remaining tumors. This signature correctly classified 12 out of 13 of the tumors of the testing set; the remaining tumor (the sporadic tumor PTC18) was unclassified but not misclassified (Figure 3, Table S2B).

### EMts_2PCA analysis of published radiation-induced breast tumor series

**Figure 4:**
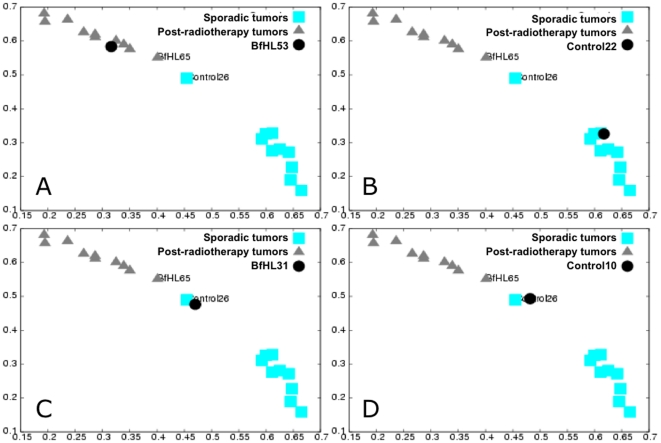
RMS classification of the testing tumors: sporadic and post-radiotherapy breast cancer. Testing tumor (sporadic breast cancers (control22 and control10)) and post-radiotherapy breast cancer (BfHL53 and BfHL31) (dot) classification considering all eigenvectors (validation space) in the RMS computation. Scatter plot of RMS_sporadic_^matrix^ as a function of the RMS_post-radiotherapy_^matrix^ for the learning tumors and the RMS_sporadic_^class^ as a function of the RMS_post-radiotherapy_^class^ of the considered testing tumor. The RMS values of the training tumors fall into two distinct groups (control sporadic breast tumors, square and post-radiotherapy breast tumors, triangle). **A**, **B**: examples of correctly classified tumors, BfHL53 and control22, respectively. **C**, **D**: outlier classification of the BfHL31 and the control10, respectively. BfHl65 and Control26 are the two learning tumors that delineate their respective validation space.

We chose this other series of 42 sporadic or post-radiotherapy breast cancers because the authors using the SAM classification (a method that was not used by Detours *et al.*), misclassified five tumors [13]. A signature of 44 genes (45 probes) was found by applying the EMts stage to a learning/training set of 20 tumors (Table S4). The robustness of this signature was assessed by the case-by-case 2PCA classification of an independent testing set composed of the 22 remaining tumors. Twenty of the 22 testing tumors were correctly classified and 2 tumors (a post-radiotherapy tumor, BfHL31 and a sporadic tumor, control 10) were unclassified but not misclassified (Figure 4, Table S2C).

### EMts_2PCA classifier efficiency

With the EMts_2PCA method the predictive classification is done according to three categories, the two groups of samples (for example PTC and FTA) and the third category corresponding to the unclassified samples. The commonly used sensitivity and specificity evaluators are poorly adapted to EMts_2PCA, because these evaluators required a dichotomous classification (2 categories). Consequently, in order to make a comparison with the performance of previously published classifiers [12], [13], we have adapted the EMts_2PCA classification into two categories: first correctly classified and second misclassified and unclassified (Data S1). It can be seen that the EMts_2PCA method is rather efficient in predicting tumor classification since, regarding the three series of tumors, the sensitivity ranged from 0.86 to 1 and specificity was always equal to 1. However, as a function of the category taken as a positive reference (Data S1), the test could give asymmetric results.

### Comparison of EMTS_2PCA with gene expression bar code, top-scoring pair(s), and PCA methods

We decided to compare the EMts_2PCA with three efficient methods for the classification of large series of samples: gene expression bar code [14], top-scoring pair (TSP) [15] and a PCA-based method [16]. We chose these three methods of classification because 1) like the EMts_2PCA, TSP uses a strategy of gene selection, without, however, a training step, 2) the bar code, in contrast, is based on a global approach without stringent criteria for the gene selection, and 3) PCA is frequently used to classify samples with or without a gene selection step.

The comparison of the three methods was done for the three series of tumors and focused on the performance of 1) the predictive classification of testing tumors and 2) all tumors clustering, without considering learning and testing groups. However, in order to better evaluate the efficiency of tumor classification, with the distinction of unclassified and misclassified tumors, and without any asymmetry as a function of the group taken as a reference (positive group), we calculated a relative predictive efficiency (RPE) defined by the number of correctly classified tumors (whatever the groups) divided by the number of predicted tumors (correctly and incorrectly predicted), and a general predictive efficiency (GPE) defined by the number of correctly classified tumors divided by the total number of tumors (correctly classified, misclassified and unclassified) (Table 1; Data S1). The high RPE and GPE scores of EMts_2PCA, whatever the series of tumors, illustrate its high efficiency in tumor prediction and its high frequency in providing a prediction.

**Table 1:** Efficiency of the blind classification of the three series of tumors by EMts_2PCA.

	EMts_2PCAP prediction			Score of the classifier	
Groups	Well	False	?	RPE	GPE
**FTAs**	15	0	0	1	1
**PTCs**	13	0	0		
**Radiation-induced breast tumors**	11	0	1	1	0.9
**Sporadic breast tumors**	9	0	1		
**Post-Chernobyl thyroid PTCs**	5	0	1	1	0.92
**Sporadic thyroid PTCs**	6	0	1		

RPE was defined by the number of correctly classified tumors divided by the number of predicted tumors (either correctly or incorrectly predicted) and GPE was defined by the number of correctly classified tumors divided by the total number of tumors (correctly classified, misclassified or unclassified). ?: unclassified; FTA: follicular thyroid adenoma; PTC: papillary thyroid carcinoma.

### Comparison of EMTS_2PCA with Gene expression bar code

The principle of the bar code consists in coding the levels of expression of all genes, 1 or 0, as a function of expressed or non-expressed, respectively. Then, a unique gene expression bar code is defined for each sample [14].

For each series (FTA/PTC, post-Chernobyl and breast tumors), a bar code limit (expressed/non-expressed) was determined for each gene, based on the distribution mode of expression of the gene in the learning set. Those limits were used to code all tumors of the considered series (learning and testing). Then, the bar codes (the tumors) were classified as a function of the correlation of the Euclidean distances.

#### Gene expression bar code prediction and clustering of PTC/FTA

Regarding tumor prediction, the 26 PTC/FTA tumors of the learning set were clearly separated into two groups, and 15 out of 28 testing tumors were correctly classified. Six of the remaining tumors were unclassified and 7 were misclassified (Table 2A, Figure S1A). Regarding tumor clustering, by considering all 54 tumors, whether learning or training, the tumors could be separated into two PTC or FTA subgroups with 7 errors and 2 unclassified.

**Table 2:** Efficiency of the blind classification of the three series of tumors.

A	Bar code Prediction			Score of the classifier	
Groups	Well	False	?	RPE	GPE
**FTAs**	6	5	4	0.68	0.53
**PTCs**	9	2	2		
**Radiation-induced breast tumors**	/	/	/	/	/
**Sporadic breast tumors**	/	/	/		
**Post-Chernobyl thyroid PTCs**	**/**	/	/	/	/
**Sporadic thyroid PTCs**	/	/	/		

B	First top-scoring pair Prediction			Score of the classifier	
Groups	Well	False	?	RPE	GPE
**FTAs**	5	4	6	0.5	0.35
**PTCs**	5	6	2		
**Radiation-induced breast tumors**	6	5	1	0.68	0.59
**Sporadic breast tumors**	7	1	2		
**Post-Chernobyl thyroid PTCs**	2	2	2	0.6	0.46
**Sporadic thyroid PTCs**	4	2	1		

C	Top-scoring pairs Prediction			Score of the classifier	
Groups	Well	False	?	RPE	GPE
**FTAs**	7	1	7	0.93	0.50
**PTCs**	7	0	6		
**Radiation-induced breast tumors**	6	0	6	1	0.50
**Sporadic breast tumors**	5	0	5		
**Post-Chernobyl thyroid PTCs**	1	1	4	0.83	0.38
**Sporadic thyroid PTCs**	4	0	3		

D	PCA Prediction			Score of the classifier	
Groups	Well	False	?	RPE	GPE
**FTAs**	10	0	5	1	0.76
**PTCs**	12	0	1		
**Radiation-induced breast tumors**	5	0	7	1	0.50
**Sporadic breast tumors**	6	0	4		
**Post-Chernobyl thyroid PTCs**	4	0	2	1	0.38
**Sporadic thyroid PTCs**	1	0	6		

Efficiency of the blind classification prediction of the three testing series of tumors by the gene expression bar code (**A**), by the first top-scoring pair (**B**), by the top-scoring pairs (**C**) or by PCA (**D**). RPE: relative prediction efficiency, GPE: general prediction efficiency (see Data S1).

#### Gene expression bar code prediction and clustering of breast tumors and Chernobyl cancers

The bar code approach did not permit classification into two subgroups of either the 20 breast learning tumors or the 13 post-Chernobyl learning tumors (Figure S1B–C). When considering all tumors, whether learning or training, tumors of both series could not be separated into two groups because they were randomly distributed (Figure S1B–C).

In conclusion, the bar code method was only efficient in separating the two groups of 26 learning PTC/FTA tumors, and was inefficient in predicting the testing tumors of this series and in either prediction or clustering of the two smaller series of breast and post-Chernobyl tumors.

### Comparison of EMTS_2PCA with top-scoring pair(s) method

The top-scoring pair (TSP) classifier selects the most discriminating genes based on the relationship between the expression of a pair of genes in a tumor (expression of gene 1> expression of gene 2). The more frequent pairs of genes that follow the relationship within a subgroup and the reciprocal relationship in the second subgroup form the TSPs [15].

The TSP method was applied to the learning set of tumors of each series (PTC/FTA, post-Chernobyl, and breast) to determine the top genes pairs with the highest frequencies. The selection of the first TSP can be used to classify the tumors. Indeed, the classification can also be made with the TSPs but this necessitates doing a PCA.

#### First top-scoring pair prediction and clustering of PTC/FTA, post-Chernobyl and breast tumors

For all three series, the learning set could be separated into two groups by using the first top-scoring pair of genes (Figure S2A–C). However, the predictive classification of the testing tumors gave poor results for the three series, even using PCA to find the best projection to maximize asymmetry (Table 2B; Figure S3A–C). Moreover, regarding tumor clustering, when considering all tumors (whether learning or testing), the first top-scoring pair did not differentiate the two subgroups of tumors (Figure S3A–C).

#### Top-scoring pairs prediction and clustering of PTC/FTA, post-Chernobyl and breast tumors

Using the learning set of tumors, the TSPs are composed of 30, 55 and 860 pairs of genes for PTC/FTA, breast cancers and post-Chernobyl tumors, respectively. These signatures were used to classify all tumors of each series using conventional PCA. As shown in (Figure S3D–F), the use of the TSPs distinguished the two subgroups of learning tumors for each three series. Regarding the predictive classification of the testing PTC/FTA, breast and post-Chernobyl tumors, they correctly predicted 14 of 28, 11 of 22 and 5 of 13 tumors, respectively. Except for two tumors misclassified (one in each FTA/PTC and post-Chernobyl series), all remaining tumors were unclassified (Table 2C; Figure S3D–F).

Regarding all tumor clustering, whether learning or testing sets, except for the post-Chernobyl series, which could not be separated into two subgroups, breast and FTA/PTC series were well clustered into two groups (only one tumor was misattributed in the FTA/PTC series) (Table 2C; Figure S3D–F).

### PCA prediction and clustering of PTC/FTA, post-Chernobyl and breast tumors

To compare the EMts_2PCA performance with that a classic PCA, we used the final signature from the EMts stage to classify all tumors of each series with a conventional PCA (Figure S3G–I).

It can be seen that the three learning sets of the three series of tumors are well separated into two groups. Regarding the prediction of the testing PTC/FTA, breast and post-Chernobyl tumors, PCA correctly predicted 22 of 28 tumors, 11 of 22 and 5 of 13 tumors, respectively (Table 2D; Figure S3G–I), all remaining tumors being unclassified. Regarding the clustering of all tumors, (whether learning or training), all series were well clustered into their respective subgroups (Figure S3G–I).

### Comparison of the performances of the EMts_2PCA method with the different tested classifiers

The barcode and TSP methods (using the first top scoring pair) are poorly adapted to tumor prediction and classification when using small learning set of tumors, as illustrated by the low RPE and GPE scores (Table 2A–B; Table S5).

Regarding tumor clustering, PCA and EMts_2PCA were the more efficient for all three series (Tables 1 and 2D; Table S5). The TSP method (using all top scoring pairs) well clustered PTC/FTA and breast tumors, but was less effective with the smaller post-Chernobyl series (Table 2C; Table S5).

Regarding the predictive classification, the TSP method (using all top-scoring pairs) and PCA gave high RPE scores, in the range of those obtained with EMts_2PCA (Tables 1, 2C–D). However, the GPE scores of the TSP method, by using all TSPs, and of PCA were rather low because of the high proportion of unclassified tumors (Table 2C–D). In comparison, the higher GPE scores of EMts_2PCA (for the three series) indicated that EMts_2PCA correctly predicts tumor classification with a higher frequency (Table 1).

## Discussion

The aim of this work was to develop a two-stage approach, based on transcriptomic analysis, to search for a specific signature discriminating two subgroups in small series of biological samples (EMts stage). Once the signature was found, it was then used to blindly diagnose new samples case by case (2sPCA stage). For this purpose, a few methods already exist [6], [10], [14], [15], but they can be inaccurate in the case of small series with high sample heterogeneity or if the gene expression signature is masked. As an example, support vector machine classification, generalized partial least-square, random forest classification, significant analysis of microarray, voting machine, hierarchical clustering, bootstrapping… were applied to the set of sporadic PTCs and post-Chernobyl PTCs, and were inefficient in finding a stable signature able to classify the tumors [11], [12]. However, the authors of these studies used an arbitrary signature, related to the human lymphocyte stress response, to classify a set of 26 tumors [12], [18]. Although these signatures misclassified some tumors, one may suspect the existence in the gene expression profiles of masked discriminating information, which was not found by the classic methods. The strategy presented in this paper was applied to this series of 26 tumors. In contrast to the results obtained with the classic methods used in the publications of Detours *et al.* [12], the EMts_2PCA method identified a molecular signature using a subset of 13 tumors (Table S3), and this signature was robust enough to classify unambiguously 12 of the 13 remaining tumors in either the sporadic PTC or post-Chernobyl PTC subgroup. The thirteenth tumor (a sporadic PTC) clustered between the two subgroups and could not be classified, but it was not misclassified as radiation-induced (Table 1; Table S2B).

As shown by using the data set of Detours *et al*., the use of an EM algorithm, combined with small combinations (5 *vs.* 5 tumors) with less than 50% tumor overlap, allowed high-probability selection of candidate marker genes taking into account the heterogeneity of gene expression within each subgroup of tumors. This point is important, since failure to take this heterogeneity into account reduces the probability that a selected gene is relevant for classifying new tumors, and also potentially eliminates the selection of numerous relevant genes. These two limitations are stronger when there are few tumors in the learning set, as the number of genes selected by chance increases. The alternative of increasing the number of tumors in the learning set to the detriment of the validation reduces the robustness of the validation. A solution often employed is to increase the size of the training set artificially by isolating by permutation each tumor and classifying it using the signature defined with the remaining tumors [9]. This solution could be problematic in establishing a relevant signature applicable to new tumors, since it may exclude the heterogeneity of expression between the tumors when the overlap is too great from one permutation to another [9]. Moreover, increasing the number of learning tumors in the expression matrices, a strategy often employed, could reduce the probability of finding genes whose expression is similar (in absolute value and sign) between the tumors of a given subgroup if the method does not take advantage of the heterogeneity.

One way to overcome too large an overlap between matrices is to use a random selection of tumors in the learning group (bootstrapping) to find sets of genes able to classify the remaining tumors [19]. A gene is kept in the final stable signature if it is present in most of the signatures that classify a tumor correctly. However, not all the classifying sets of genes are necessarily found, and the extreme spatial localization of the tumors of certain random selections can lead to sets of genes that do not discriminate tumors of median localization [20]–[22]. The impossibility of finding a robust final signature can therefore result from an insufficient exploration of the space. Consequently, failure to find a robust signature does not necessarily mean that there is none. To increase the probability of finding a robust signature, a gene must not be selected solely as a function of the number of candidate signatures in which it has been found, but essentially on the basis of its capacity to play a part in classifying tumors. The training step introduced in our method is more appropriate for evaluating the capacity of each gene to classify a tumor through its frequency of occurrence in the training matrices.

The critical point now is to identify among the candidate genes those with the greatest potential to classify new tumors. For this, we define a relevance factor (Material and Methods: Search for the final signature, Compilation) calculated from each training tumor classification. For each gene, we thus obtain an objective criterion for assessing the probability that it will classify a tumor not used for its selection. However, selection of genes, despite heterogeneity in their expression within a given subgroup, does result in scattering of the tumors in the N-dimensional space defined by the selected genes. This scatter around a mean position, or entropy, increases with the number of tumors. Mathematical treatments, such as centering and normalization of the matrices, also modify the entropy and therefore the relative positions of the tumors. This happens not only as a function of the composition of the combinations, but also of the tumors to be classified. To minimize entropy and to compare centered and normalized expressions between all the combinations, the expressions of all tumors (in the learning and validation sets) are standardized. The standardized expression values projected into the validation space lead to constant distribution of the learning tumors, allowing the relative positioning in the validation space of new tumors to be classified. This standardization strategy was applied with success on data from microarray of same technology. However, the mix of data from microarrays with different technologies could increase the entropy of the results because of potential differences in the background noise and/or scale range of the measurements.

After the first result obtained with the post-Chernobyl dataset, which underlined the high potential of our strategy for case-by-case blind diagnosis of small series of radiation-induced thyroid tumors (Table 1), this strategy was applied to another series of breast cancer tumors, either sporadic or induced after radiotherapy, already analyzed and for which the authors classified the tumors with 5 errors using the SAM method [13]. A discriminating 44-gene signature was identified from a learning/training set of 20 tumors (Table S4). This signature correctly classified 20 of 22 independent testing tumors and misclassified no tumor, since the 2 remaining tumors clustered between the two groups (Table 1; Table S2C). These data showed again that, as for the methods used for post-Chernobyl tumor classification, the EMts_2PCA method was more efficient than SAM. Finally, the EMts_2PCA method was also applied to the two most frequent histological types of thyroid tumors (FTA and PTC), which are studied in the laboratory [8] and for which there are numerous data in the literature [8], [19], [23]–[25]. This method was used to define a discriminating 227-gene signature from a learning/training set of 26 tumors (Table S1). This signature correctly blindly diagnosed the histology of 28 independent testing tumors (Table 1; Figure 2; Table S2A). To validate the biological relevance of the signature obtained with this new method, we first checked its overlap with 50 published thyroid tumor transcriptomic analyses. Among the genes that have been shown to differentiate histological subtypes of thyroid tumors and/or to be associated with the expression of thyroid oncogenes (RET/PTC isoforms or BRAFV600E), 78 genes overlapped with the present signature (Table S1). Notably, 39 genes in this overlap have already been identified in signatures discriminating malignant thyroid tumors (PTC, follicular variant of PTC and/or follicular carcinomas), from benign thyroid tumors (FTA, hyperplastic nodules). Genes identified in the signature were relevant to molecular mechanisms associated with thyroid physiology or thyroid tumorigenesis (data S2).

The validity of a transcriptomic signature could be a matter of discussion according to the size of the series of tumors, but it is important to make a distinction between the size of the series used for the learning and the testing [26]. In any case, the robustness of a gene signature should be evaluated only on the score of the classification and on the number of tumors used in the testing set. Our strategy blindly predicted 28 FTA and PTC thyroid tumors, 22 sporadic and post-radiotherapy breast tumors and 13 post-Chernobyl and sporadic PTCs with a sensitivity of 1, 0.9 and 0.86, respectively, and a specificity of 1 for the three series (data S1), which are good scores as compared with published data [12], [13], [26]. It should be mentioned that the number of blindly classified tumors in our study is in the range of the number of tumors used for the blind validation in other studies [26]–[28].

To complete the positioning of the EMts_2PCA method, we compared its performance in classifying the three series of tumors with three other methods of tumor classification: the gene expression bar code method [14], the top-scoring pair (TSP) classifier method [15] and the PCA method [16]. In order to compare the different methods in terms of overall classification, without definition of a reference group (as required for sensitivity and specificity calculation), we defined RPE and GPE evaluators, which indicate the reliability and the effectiveness of a method in providing a prediction, respectively. These evaluators are tools used to assess the performance of classification methods without choosing a positive test as a reference, and can be used when the test is based on more than two categories.

The bar code appeared to be the less robust method for analyzing small series of samples as shown by the RPE and GPE scores (Table 2A; Table S5): it only classified/predicted the PTC/FTA series, but with errors. The bar code method is rather well adapted to large series of samples [29], and/or to samples that could be discriminated by first-order information, as illustrated by the near perfect classification of normal versus cancer [14], and of benign FTA versus malignant PTC tumors in the present work. One explanation could be that either the first-order information and/or the high number of tumors in each learning group converges toward a better estimation of the code of each gene, and thus toward a better estimation of the bar codes of the tumors, which leads to successful tumor classification. When the number of learning tumors decreased, the proportion of stochastic measures of gene expression increased, and thus the gene codes are biased by noise or chance. To avoid a selection of genes presenting a sporadic expression in the final signature, the EMts_2PCA method evaluates, in the training step, the capacity of the genes to classify the training tumors. Moreover, the EMts_2PCA method maximizes the signal on which the classification is focused (for example, FTA/PTC), even if this signal is not first order, and minimizes the other signals from potential confounding factors (for example, etiology, age, grade). For that, specific attention was paid to the choice of the learning set of tumors in order to build permutation matrices such that each half-learning matrix is homogeneous only for the criteria of classification and heterogeneous regarding the confounding factors (Material and Methods: Learning step/Search for set of candidate genes). This strategy leads to the selection of the genes informative for the sought criteria. Moreover, the stage of compilation and standardization of gene expression (Material and Methods: Search for the final signature) maximizes the discrimination.

Regarding the TSP classifier, as illustrated in (Figure S2 and S3A–F), both the first top-scoring pair and the top-scoring pairs efficiently separate the three learning sets of tumors into two groups (Table S5). If only the first top-scoring pair is considered, tumor prediction ability decreased greatly with low RPE and GPE scores (Table 2B). By contrast, if all top-scoring pairs are used, the predictive classification is rather good, as illustrated by the good RPE scores, which are lower but close to those obtained with EMts_2PCA, the GPE scores being, however, relatively low (Tables 1 and 2C; Figure S3D–F). Overall, top-scoring pairs gave good results when a tumor is classified within a group, but many tumors are unclassified. Consequently, even if the use of top-scoring pairs is robust in searching for genes able to cluster small series of samples, the prediction is less efficient than that of EMts_2PCA. One explanation could be that TSP classifier gene selection includes neither the training step nor the maximization of the information on which the classification is focused. In that context, to compare the performances of the final signature versus the gene selection using the top-scoring pairs, and to confirm the gain of the standardization and normalization steps, we used the final signature with conventional PCA, instead of the 2PCA stage, for the classification of the three series of testing tumors. For predictive classification using conventional PCA, the final signature gives similar (breast series) or better (PTC/FTA and post-Chernobyl series) performances than top-scoring pairs of genes. However, the performances are always worse than those obtained with the 2PCA stage (Table 2C–D; Figure S3D–I). So, EMts_2PCA is more appropriate in finding a signature in small series of samples, the compilation and standardization steps increasing the power of the signature in discriminating the two groups. However, when dealing with large series of samples, the use of the top-scoring pairs of genes is robust enough to identify a set of discriminating genes and thus minimizes the need for a training step. Moreover, as compared with a classic PCA method, which requires an arbitrary decision of the experimenter to assign a tumor to one group or another, EMts_2PCA permits an automatic and objective training tumor group attribution, according to the RMS scores (Material and Methods: Methods of classification by Two-step PCA and aid to classification), thus decreasing the number of unclassified tumors (Tables 1, 2D).

Interestingly, when comparing the three different methods, gene expression bar code, TSP and EMts_2PCA, the number of selected genes in the signature ranged from a pair of genes (TSP) (if a unique pair of genes presents the highest frequency), nearly all genes (bar code), to from a few dozen to a few hundred (EMts_2PCA and TSP). We considered that the impact of the number of genes should be separately discussed as a function of the aim of the analysis, either tumor clustering or tumor prediction. As illustrated in the present work, TSP always permitted clustering of the learning tumors of the three series, whatever the number of pairs of genes (from one to a few hundred) (Figure S2, S3A–F and Table S5). Regarding the prediction, it was not efficient for the three series when the first pair of genes was applied, but became efficient when all the selected top-scoring pairs were used. It should be noted that the number of genes pairs selected by TSP was higher in the smaller learning sets, but the RPE and GPE scores were high whatever the number of selected pairs of genes. Regarding the tumor prediction, no one can prejudge the number or exact selection of the genes that may be necessary for an accurate predictive classification, and this is obviously very dependent on the homogeneity/heterogeneity of the series of tumors. For example, TSP, which gives high scores for tumor prediction, does not search for the minimal number of genes necessary for tumor prediction, but selects all pairs of genes presenting individually a high score for tumor classification, without estimation of the global potential of the selection of genes for tumor prediction [15]. In this context, the EMts_2PCA method does not arbitrarily fix the minimal number of genes that should be included in a signature, but selects, through the training matrices, the genes with higher potential for predictive classification according to the rule: “at least one training tumor should be correctly classified and none should be misclassified” (Material and Methods: Training step with internal cross-validation and Compilation). With the EMts_2PCA method, no relation could be made between the number of learning tumors, the number of genes in the final signature and the performance in predictive classification, which is mainly dependent on the series of tumors.

In conclusion, the EMts_2PCA method is a strategy especially dedicated to identification, in small series of samples, of a gene expression signature discriminating subgroups of tumors, which can be applied to predict the classification of prospective tumors for diagnostic purposes. This strategy includes a standardization process to maximize the signal on which the classification is focused and defines a validation space permitting precise relative positioning of prospective tumors case by case. Importantly, this strategy allows an increasingly accurate and robust definition of the populated area of the validation space, as the number of diagnosed tumors increases, leading to a better diagnostic tool. This strategy was successfully used to blindly classify a set of thyroid cancers for which a biologically relevant signature was found and two series of post-radiotherapy and post-Chernobyl tumors for which we identified a molecular signature presenting higher scores in terms of sensitivity and specificity than those reached with the most commonly used methods of tumor classification.

## Materials and Methods

### Tissue samples

Fifty-four tumor samples (28 follicular thyroid adenomas (FTAs) and 26 papillary thyroid carcinomas (PTCs)) were obtained from the Gustave Roussy Institute and from the human BioBank in Nice (Cancéropole PACA and CRB INSERM, CHU Nice) [30], [31]. 13 FTAs and 13 PTCs were used as a learning/training set, and the remaining tumors were used as independent tumors in a testing set. Tissues were harvested immediately on arrival at the pathology suite, placed in liquid nitrogen and stored at −80°C until used. Pathological diagnoses were performed according to WHO guidelines.

Patients hospitalized at the Pasteur Hospital (Department of Otorhinolaryngology, Nice, France) gave their signed agreement to participate in the study. The protocol was approved by the local ethics committee of the University of Nice (“Comité de Protection des Personnes” and the DRCVI of the CHU of Nice) and by the French Ministry of Research (N°DC-2008-391 and N°AC-2008-83). Written Informed consent was obtained from all patients of the Institut Gustave Roussy and the study was performed in accordance with protocols previously approved by the ethics committee of Bicêtre and by the institutional review board of Institut Gustave Roussy.

### RNA extraction, labeling and hybridization

RNA samples were prepared and hybridized as described in [31], [32]. All samples were hybridized on human 25 K 50–52 mer oligo microarrays (Resogen Program, RNG/MCR, Evry), [33]. Each tumor sample was co-hybridized with a common pool of amplified normal thyroid, used as reference, and all hybridizations were duplicated in dye-swap.

### Microarray analysis

After hybridization, each spot was defined automatically using image-analysis spot-tracking software (US patent 10/173,672; CA 2,389,901). Fluorescence intensity values for both dyes (Alexa Fluor® 555 and Alexa Fluor® 647) and local background-subtracted values for individual spots were obtained using an EM algorithm (US patent 10/173,672; CA 2,389,901). Data normalization was performed as described previously [8]. Microarray data were generated in a MIAME compliant format and raw data were deposited in the Array Express database (Accession number E-MEXP-2335).

### Published thyroid and breast data

The whole procedure was also applied to previously published datasets, generated in a MIAME compliant format.

The thyroid dataset (Accession number GSE3950) was previously analyzed to identify gene expression profiles in sporadic and post-Chernobyl PTCs [12]. The initial matrix was composed of 14 sporadic PTCs, 12 post-Chernobyl PTCs and 12 429 genes. Due to missing data, 7898 of the 12 429 genes were analyzed in the present study. A learning/training set of 7 sporadic and 6 post-Chernobyl PTCs was built to avoid any bias due to age at diagnosis or sex of the patients or to genetic alterations in the tumors, the remaining tumors (7 sporadic PTCs and 6 post-Chernobyl PTCs) being used as a testing set.

The breast tumor dataset (accession number E-NCMF-30) was previously analyzed to identify gene expression profiles in sporadic and post-radiotherapy breast tumors [13]. The initial matrix was composed of 20 sporadic tumors, 22 post-radiotherapy tumors and 34 975 probes. A learning/training set of 10 sporadic and 10 post-radiotherapy tumors was built, to avoid any bias due to grade or to genetic alterations in the tumors. The remaining tumors (10 sporadic and 12 post-radiotherapy tumors) were used as a testing set.

Description of tumor samples, microarray hybridization and processing were reported in the respective publications.

### Method: Process of learning/training with internal cross-validation

This is a new two-stage strategy for tumor classification called EMts_2PCA, which comprises:

1. a first stage, named EMts, composed of 3 steps: learning, training, and search for a final signature,
2. a classification stage, called 2PCA.

The method was developed using GCC language and for the moment is not ready for use. Further time is needed for this, but we are fully willing to analyze data on request.

The method is presented taking the FTA/PTC series as an example.

#### Learning step/Search for set of candidate genes

The learning set included two subgroups of distinct histology (13 FTAs and 13 PTCs). To avoid bias between subgroups in the gene selection, the tumors of each subgroup were chosen to be comparable between the subgroups in terms of gender and age of patients at tumor diagnosis. The learning tumors were chosen to be similar between the two groups for all parameters other than the classification focus parameter.

The learning/training process is based on the analysis of combinatorial matrices of 10 tumors composed of 5 tumors of one group (half-matrix) and 5 tumors of the other group. For each histological subgroup, all the half-matrices of a combination of 5 in 13 were built. The FTA and PTC series included tumors of two different etiologies: sporadic or radiation-induced. To avoid intra-subgroup bias due to the etiology, all half-matrices containing only tumors with the same etiology were eliminated. Finally, we kept only the half-matrices that differed by more than 50% in histological composition. These selected half-matrices were used to build all possible combinatorial 5-FTA half-matrices versus 5-PTC half-matrices. Considering all these rules for keeping the matrix and building the learning set, five tumors per half-matrix were chosen because it is the smaller size of half-matrix for which each learning tumor is present at least in one of the selected 10-tumor matrices. In each resulting 10-tumor matrix, a randomized t-test was applied as a quick filter to discriminate differentially expressed genes from those belonging to the noise. The cut-off for considering that a gene is differentially expressed is the p value of the randomization t-test calculated for a noise model based on all permutations between the two groups of tumors [17]; a gene is selected if its p value is less than the cut-off.

Each 10-tumor matrix, filtered by the cut-off, was centered and normalized. Then, an EM algorithm, based on a melting model (US patent 10/173,672; CA 2,389,901) [32], was used to search for differentially expressed genes (opposite ways) to discriminate the two subgroups in a given 10-tumor matrix [34], [35]. The EM algorithm was applied to each resulting 10-tumor matrix to calculate a probability, which was derived from the weighted voting method [6] and from the method described by Chevillard et al. [8]. For each gene expression value, the EM algorithm calculated two probabilities P*_over_* and P*_under_* to be over- and under-expressed (where P*_over_*+P*_under_*. = 1), respectively, taking into account the overall gene expression values of the matrix. To be eligible as a candidate gene, a gene 1) must have for each of its expression levels in each tumor a probability difference defined as (1−|(P*_under_*−P*_over_*)|) less than 0.05, and 2) must have its expression values always deregulated in the same way (induced or down-regulated) in a given tumor group and in an opposite way (down-regulated or induced, respectively) in the other group. Finally, each 10-tumor matrix was restricted to its own selected candidate gene to build the training matrices.

#### Training step with internal cross-validation

Each training matrix was used to classify by a two-step principal component analysis (2sPCA) the remaining 16 tumors of the learning set (training tumors) (Material and Methods: Methods of classification by Two-step PCA and aid to classification, Classification of training tumors). Then, rules for tumor classification were applied. If at least one training tumor was correctly classified and none was misclassified, the training matrix was retained; otherwise it was discarded. The operation was renewed for each training matrix. The process continued if at least 90% of the training tumor classifications were validated by at least one of the training matrices. In these conditions, 10% of the tumors may not be validated, but none must be rejected by the retained training matrices (validated training matrices).

### Method: Search for the final signature

#### Compilation

The frequency of relevance F(i,j) of each gene (i) for each tumor (j) represents the frequency at which the gene (i) : -row (i), and the tumor (j) :-column (j), are found together in a validated training matrix, weighted by the number of training tumors correctly classified by this training matrix, such that:
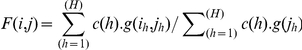
where H represents the total number of validated training matrices, (i_h_) and (j_h_) represent the coordinate of a gene and of a tumor in a given training matrix corresponding to the same gene and tumor indexed by (i) and (j) in the frequency F matrix. For each training matrix, g(i_h_,j_h_) = 1 or 0 depending on the gene and the tumor's presence or absence, respectively, and g(j_h_) = 1 or 0 depending on the tumor's presence or absence. Where c(h) = number of training tumors correctly classified by the training matrix (h).

The mean frequency of relevance F(i) of a gene was defined as:
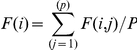
representing the average of the row (i) of the matrix F(i,j), with P the total number of tumors in the learning set.

The mean frequency of relevance F(i) of a gene is the frequency by which the gene participated successfully in the classification of the training tumors through all training matrices.

The mean marginal frequency F(j) of a tumor is defined as:
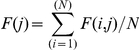
representing the average of the column (j) of the matrix F(i,j), with N the total number of candidate genes considering all training matrices.

The mean marginal frequency F(j) was the mean frequency with which any candidate gene successfully participated in the classification of the training tumors through all training matrices.

The mean of F(j) was defined as:
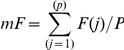
The standard deviation of F(j) was defined as:
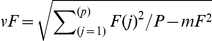
with P the number of tumors in the learning set.

A gene (i) was kept for the final signature used to classify the testing set if F(i)≥*m*F+2*v*F, where *m*F+2*v*F is the threshold from which the cumulative distribution function is asymptotic to 1 and thus the threshold where F(i,j) becomes statistically singular.

#### Standardization

The mean m_h_(i_h_) and the module M_h_(i_h_) of each line of the training matrix were calculated such that:
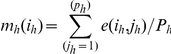
and
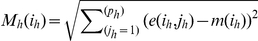
where each selected training matrix e_h_ comprises in line (i_h_) the (n_h_) candidate genes, and in column (j_h_) the (p_h_) tumors.

Each training matrix e_h_ was then completed by the gene expressions of the corresponding candidate genes of the training tumors (tumors of the learning set absent from the considered training matrix) and of the testing tumors, to obtain a matrix e′_h_ of L = 55 tumors.

Each line of a matrix e′_h_ was then centered such that:
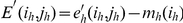
and normalized such that:
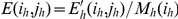
All matrices E_h_ were compiled in a single matrix in which a member ξ(i,j) represented the centered and normalized mean of the expression E_h_ (i_h_,j_h_) of this same gene I_h_ and of this same tumor j_h_ in all matrices E_h_:
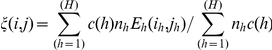
with i and j the respective coordinates of a gene and of a tumor in ξ and i_h_,j_h_ the respective coordinates of the same gene and of the same tumor in E_h_. The matrix ξ was restricted to the genes of the final stable signature.

### Methods of classification by Two-step PCA and aid to classification

#### Classification of training tumors

The eigenvectors and eigenvalues of a training matrix were calculated. A Cartesian coordinate system was defined from the three eigenvectors with the largest eigenvalues. Then, all tumors of this training matrix and each corresponding training tumor were projected onto the Cartesian coordinate system (training space), which specifically maximized the asymmetry between the training matrix tumors. The positioning of each training tumor in the Cartesian coordinate system, compared with the positioning of the training matrix tumors, enabled the training tumor to be classified as FTA or PTC.

#### Classification of testing tumors

The eigenvectors and eigenvalues were calculated for the matrix ξ′(i,j) from the ξ(i,j) restricted to the learning set tumors. These vectors define a new space maximizing specifically the asymmetry between the learning tumors. A new matrix of coordinates Ψ(i,j) was then calculated for all tumors of the testing and learning sets by projection of vector columns (i) of the matrix ξ(i) onto the eigenvectors. A Cartesian coordinate system was defined from the three eigenvectors with the largest eigenvalues (validation space). The location of each testing tumor in the validation spaces, compared with the location of the learning tumors, enabled the testing tumor to be classified as FTA or PTC.

#### Aid to classification

In the training spaces or validation spaces, outlier tumors may have a spatial position at the border of the subgroups, meaning it may be difficult to assign them to one or another subgroup. To be more discriminatory, we propose to assess the distances precisely, taking into account more than three eigenvectors if necessary. To do so, we used a decision-making tool based on calculation of the root mean square (RMS). The RMS of a tumor (j), a function of the barycenter of the subgroup g (g = FTA or PTC) in a given matrix ϕ(i,j), is defined as
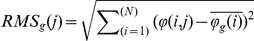
where
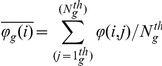
with 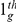 is the first and 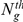 the last tumor of the subgroup g.

#### Aid to classification of training tumors

For each tumor j in a given training matrix, both RMS_FTA_(j)^matrix^ and RMS_PTC_(j)^matrix^ were calculated between each training matrix tumor j and the barycenter of the FTA and PTC subgroups, respectively. To assign a training tumor k to the FTA or PTC subgroup, RMS_FTA_(k)^class^ and RMS_PTC_(k)^class^ were calculated between the tumor k and the barycenter of the FTA and PTC subgroups, respectively.

#### Aid to classification of testing tumors

For each tumor j in the ξ′ matrix from the ξ matrix restricted to the learning set tumors, both RMS_FTA_(j)^matrix^ and RMS_PTC_(j)^matrix^ were calculated between each ξ′ matrix tumor j and the barycenter of the FTA and PTC subgroups, respectively. To assign a testing tumor k to the FTA or PTC subgroup, the RMS_FTA_(k)^class^ and RMS_PTC_(k)^class^ were calculated between the tumor k and the barycenter of the FTA and PTC subgroups, respectively.

#### Classification rules as a function of the RMS

To assign a tumor k to a given histological subgroup (for instance, FTA subgroup = given-subgroup and PTC subgroup = other-subgroup, or inversely), the RMS_given-subgroup_(k)^class^ of the tumor must be lower than at least one RMS_given-subgroup_(j)^matrix^ of given-subgroup tumors, taking into account the RMS_given-subgroup_(j)^matrix^ variance.

The classification is attributed if the tumor to be classified is correctly assigned and no tumor of a given subgroup has an RMS_given-subgroup_(j)^matrix^ more than the RMS_other-subgroup_(j)^matrix^ of one tumor of the other subgroup.

The classification is unattributed if the tumor to be classified is not assigned to a subgroup, and no tumor of a given subgroup has an RMS_given-subgroup_(j)^matrix^ more than the RMS_other-subgroup_(j)^matrix^ of one tumor of the other subgroup.

The classification is rejected if the tumor to be classified is misclassified, or if any tumor of a given subgroup has an RMS_given-subgroup_(j)^matrix^ more than the RMS_other-subgroup_(j)^matrix^ of one tumor of the other subgroup.

### Selection of eigenvectors to improve the classification (new validation space)

Eigenvectors with the largest eigenvalues define the directions of the N-dimensional space that presents the greatest asymmetry (total variance) between the tumors [36]. However, the greatest asymmetry between the tumors guarantees neither the homogeneity in expression levels of tumors of a given subgroup nor the highest asymmetry between the two subgroups of tumors. Indeed, heterogeneity of gene expression can increase asymmetry between the tumors and thus leads to entropy in the position of tumors of a given subgroup; this hampers classification of tumors borderline to the subgroup. The total variance between the tumors can be decomposed as the sum of calculated variances between the tumors of each group (Σ V_within_group) plus the variance between the centroid of each group (V_inter_groups). To reduce this entropy, the eigenvectors that jointly define the greatest asymmetries between the two subgroups of tumors and the best expression level homogeneity within a given subgroup are selected, using the ClusterIt method [8], thus maximizing the ratio V_inter-groups/Σ V_within-group.

## Supporting Information

Figure S1 **Tumor clustering and prediction using the barcode method: correlation map between the tumor barcodes.** The barcode method was applied to the 3 series of samples: series of thyroid follicular adenomas (FTAs) and thyroid papillary carcinomas (PTCs) (**A**), series of sporadic breast cancers (control) and post-radiotherapy breast cancers (BfHL) (**B**) and a series of sporadic PTCs and post-Chernobyl PTCs (**C**). In each series, the tumors of the learning set are indicated by full blue or green dots and corresponding testing samples are indicated by open blue or green circles as a function of the group in which they should be classified.
(TIF)

Figure S2 **Learning tumor clustering using the first top-scoring pair.** The figure represents the relative positioning of the learning tumors of the PTC/FTA (**A**), breast (**B**) and post-Chernobyl (**C**) series of tumors as a function the first top pair of genes. In each series, learning tumors are indicated as red or blue dots as a function of the two groups. The black line represents the decision boundary. FTA: thyroid follicular adenoma, PTC: thyroid papillary carcinoma.
(TIF)

Figure S3 **Classification of the three series of tumors using the first top-scoring pair of genes, all the top-scoring pairs and the final signature in a conventional principal component analysis (PCA).** The figure represents the relative positioning of all samples (learning and testing) of each series of tumors PTC/FTA (**A**, **D** and **G**), breast tumors (**B**, **E** and **H**) and post-Chernobyl tumors (**C**, **F** and **I**) as a function of the two eigenvectors with the highest eigenvalues using the first top-scoring pair (**A**, **B** and **C**), all the selected top-scoring pairs (**D**, **E** and **F**) and the final signature (**G**, **H** and **I**). Learning tumors are indicated as blue squares or gray triangles as a function of the group, and the corresponding testing tumors (X samples) are indicated as red crosses or orange dots, respectively. Unclassified tumors are indicated by a red ring and tumors misclassified by a red arrow. The blue line represents the frontier between the two groups of samples, when possible to do so. FTA: thyroid follicular adenoma, PTC: thyroid papillary carcinoma, S: sporadic breast and thyroid tumors; R: post-radiotherapy breast cancer or post-Chernobyl PTC.
(TIF)

Table S1 **List of genes (final stable signature) discriminating follicular thyroid adenomas (FTAs) from thyroid papillary carcinomas (PTCs).** Differential gene expression values were calculated in the validation space as the average of log (PTC gene expression) minus the average of log (FTA gene expression), with the corresponding p value. References are indicated if genes were already identified in other thyroid-associated signatures.
(DOC)

Table S2 **RMS values.** Testing tumors were classified by considering the RMS in the respective validation spaces (see Material and Methods: Aid to classification of testing tumors). The delta RMS threshold is the shortest distance between the two RMS scatter plots of the learning tumor minus the sum of the standard deviation to the barycenter of each RMA scatter plot. **A**: Thyroid tumors series (FTA vs PTC). **B**: Post-Chernobyl series. **C**: Post-radiotherapy breast cancer series. FTA: follicular thyroid adenoma, PTC: papillary thyroid carcinoma, R: radiation-induced, S: sporadic, ?: unclassified.
(DOC)

Table S3 **List of genes (final stable signature) discriminating sporadic thyroid papillary carcinomas from post-Chernobyl thyroid papillary carcinomas.** The signature was determined from a published dataset retrieved from the GEO database (http://www.ncbi.nlm.nih.gov/geo, accession number GSE3950). Differential gene expression values were calculated in the validation space as the average of log (post-Chernobyl PTC gene expression) minus the average of log (sporadic PTC gene expression), with the corresponding p value.
(DOC)

Table S4 **List of genes (final stable signature) discriminating sporadic breast cancers from post-radiotherapy breast cancers.** The signature was determined from a published dataset retrieved from the ArrayExpress database (http://www.ebi.ac.uk/microarray-as/ae, accession number E-NCMF-30). Differential gene expression values were calculated in the validation space as the average of log (control breast tumor gene expression) minus the average of log (radiation-induced breast tumor gene expression), with the corresponding p value.
(DOC)

Table S5 **Results of clustering obtained for the three series of tumors with the different methods.** Data are given in terms of either the classification of learning tumors alone or all tumors (learning and testing). Yes: indicates that the learning set/all tumors were well clustered into two groups. No: indicates that the learning set/all tumors were not well clustered into two groups. FTA: follicular thyroid adenoma; PTC: papillary thyroid carcinoma, R: radiation-induced tumors; S: sporadic tumors; n (number of gene pairs) = 30, 55 and 860 for FTA/PTC, breast tumors and post-Chernobyl series, respectively. *Only one error.
(DOC)

Data S1 **Performance of the classifier.**
(DOC)

Data S2 **Analysis of the signature discriminating thyroid follicular adenomas from thyroid papillary carcinomas.**
(DOC)
