# Supplementary figures and images for: Strategy to Find Molecular Signatures in a Small Series of Rare Cancers: Validation for Radiation-Induced Breast and Thyroid Tumors
Source: PLoS One. 2011 Aug 11;6(8):e23581. doi: 10.1371/journal.pone.0023581 (PMC3154936; doi:10.1371/journal.pone.0023581)

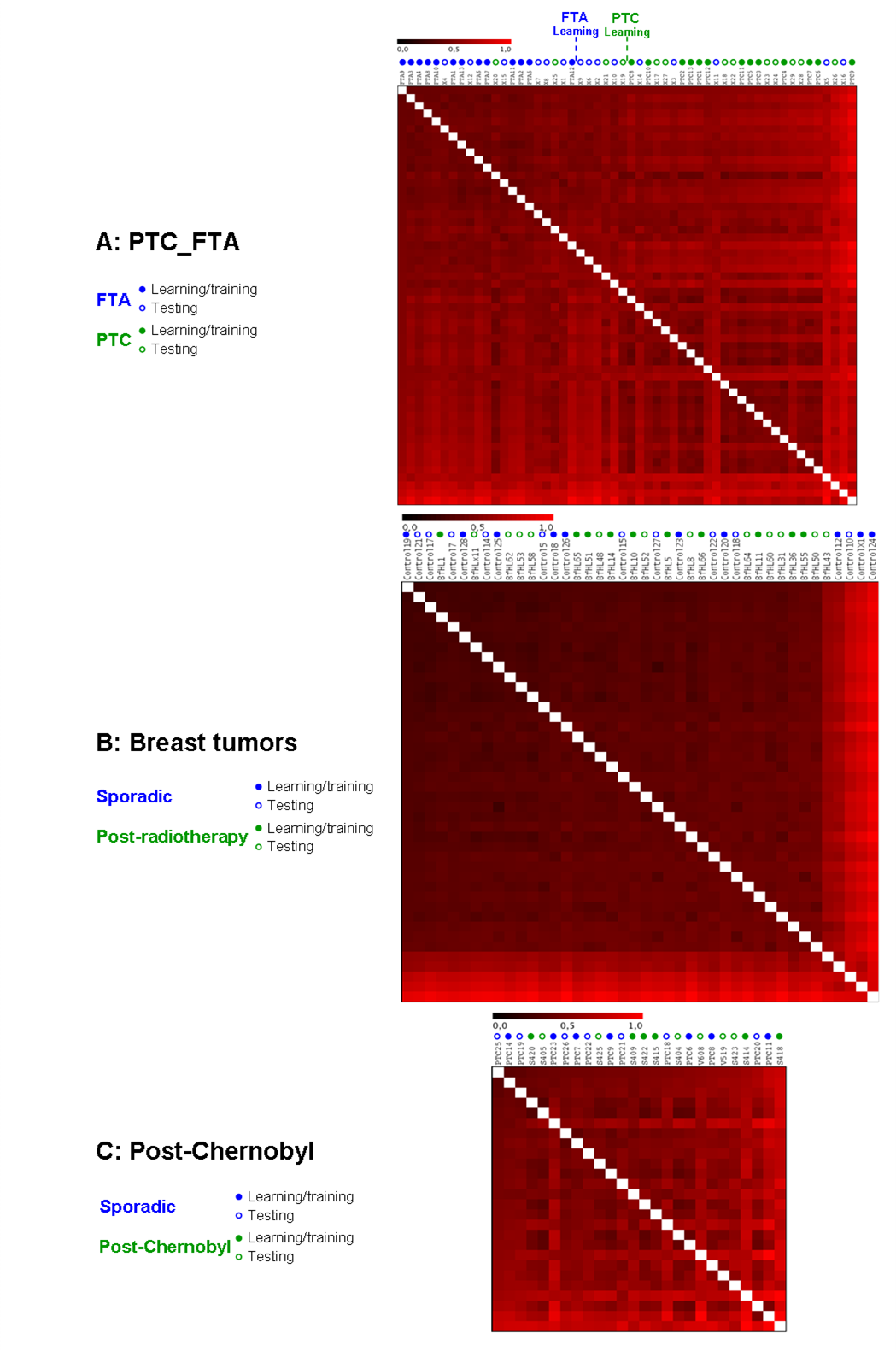

Supplement: Figure S1 — Tumor clustering and prediction using the barcode method: correlation map between the tumor barcodes. The barcode method was applied to the 3 series of samples: series of thyroid follicular adenomas (FTAs) and thyroid papillary carcinomas (PTCs) (A), series of sporadic breast cancers (control) and post-radiotherapy breast cancers (BfHL) (B) and a series of sporadic PTCs and post-Chernobyl PTCs (C). In each series, the tumors of the learning set are indicated by full blue or green dots and corresponding testing samples are indicated by open blue or green circles as a function of the group in which they should be classified. (TIF) [file pone.0023581.s001.tif]

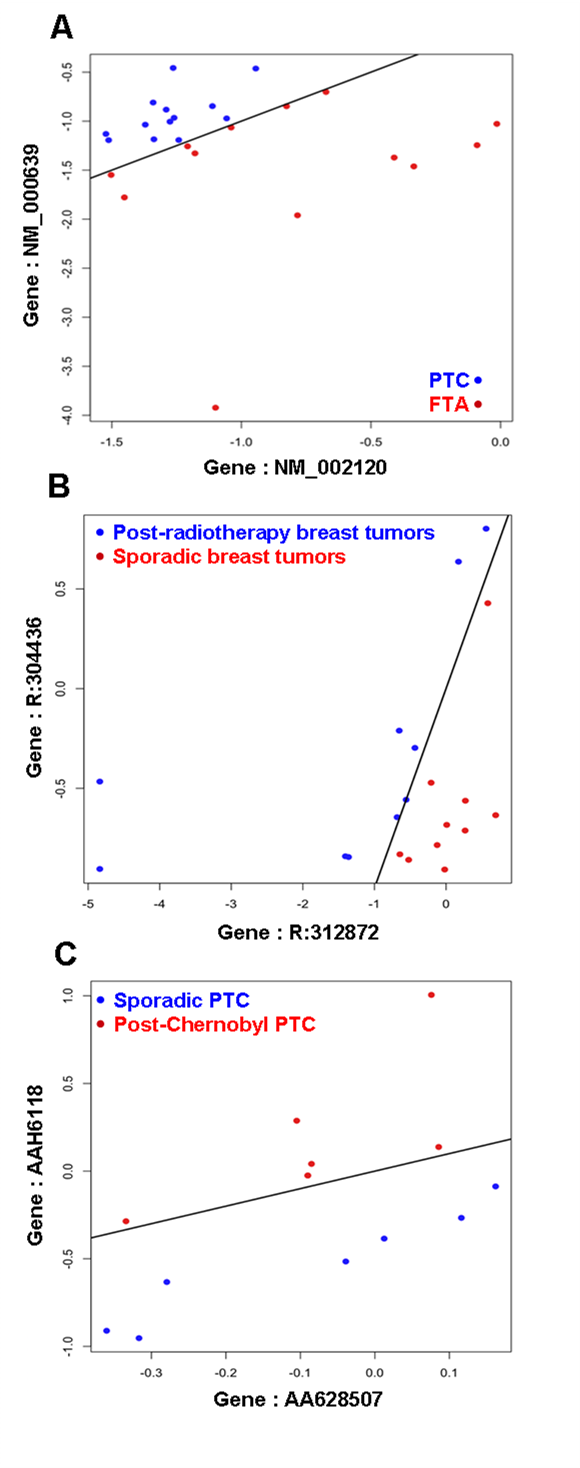

Supplement: Figure S2 — Learning tumor clustering using the first top-scoring pair. The figure represents the relative positioning of the learning tumors of the PTC/FTA (A), breast (B) and post-Chernobyl (C) series of tumors as a function the first top pair of genes. In each series, learning tumors are indicated as red or blue dots as a function of the two groups. The black line represents the decision boundary. FTA: thyroid follicular adenoma, PTC: thyroid papillary carcinoma. (TIF) [file pone.0023581.s002.tif]

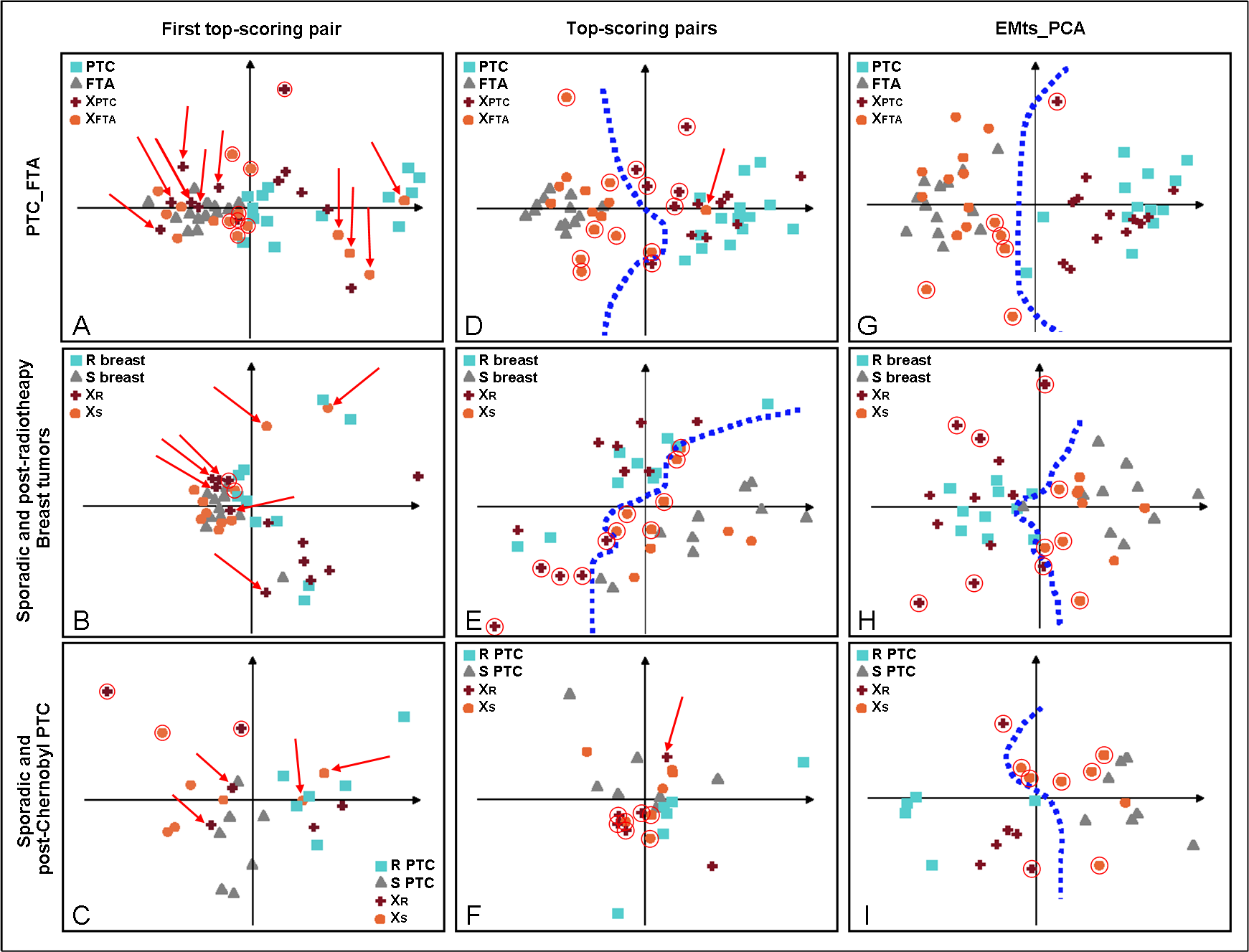

Supplement: Figure S3 — Classification of the three series of tumors using the first top-scoring pair of genes, all the top-scoring pairs and the final signature in a conventional principal component analysis (PCA). The figure represents the relative positioning of all samples (learning and testing) of each series of tumors PTC/FTA (A, D and G), breast tumors (B, E and H) and post-Chernobyl tumors (C, F and I) as a function of the two eigenvectors with the highest eigenvalues using the first top-scoring pair (A, B and C), all the selected top-scoring pairs (D, E and F) and the final signature (G, H and I). Learning tumors are indicated as blue squares or gray triangles as a function of the group, and the corresponding testing tumors (X samples) are indicated as red crosses or orange dots, respectively. Unclassified tumors are indicated by a red ring and tumors misclassified by a red arrow. The blue line represents the frontier between the two groups of samples, when possible to do so. FTA: thyroid follicular adenoma, PTC: thyroid papillary carcinoma, S: sporadic breast and thyroid tumors; R: post-radiotherapy breast cancer or post-Chernobyl PTC. (TIF) [file pone.0023581.s003.tif]
