# Supplementary material for: Strategy to Find Molecular Signatures in a Small Series of Rare Cancers: Validation for Radiation-Induced Breast and Thyroid Tumors
Source: PLoS One. 2011 Aug 11;6(8):e23581. doi: 10.1371/journal.pone.0023581 (PMC3154936; doi:10.1371/journal.pone.0023581)
Supplement: Table S1 — List of genes (final stable signature) discriminating follicular thyroid adenomas (FTAs) from thyroid papillary carcinomas (PTCs). Differential gene expression values were calculated in the validation space as the average of log (PTC gene expression) minus the average of log (FTA gene expression), with the corresponding p value. References are indicated if genes were already identified in other thyroid-associated signatures. (DOC) [file pone.0023581.s004.doc]

**List of genes (final stable signature) discriminating follicular thyroid adenomas (FTAs) from thyroid papillary carcinomas (PTCs)**

| **Accession Number** | **Symbol** | **Differential gene expression** | **p_value** | **References** |
| --- | --- | --- | --- | --- |
| **Signal transduction** | |  |  |  |
| AK124547 | **ABR** | 0.796 | 0.0032 | (9; 27) |
| AK128511 | **ADIPOR2** | -0.483 | 0.0046 |  |
| NM_032468 | **ASPH** | -0.584 | 0.0044 | (9) |
| NM_032467 |  | -0,798 | 0.0036 |  |
| NM_005188 | **CBL** | 0.450 | 0.0052 |  |
| AL832074 | **CD36** | -0.706 | 0.0049 | (22; 28) |
| NM_006449 | **CDC42EP3** | 0.561 | 0.0045 | (1) |
| BQ430655 | **DUSP23** | 0.342 | 0.0031 |  |
| NM_057158 | **DUSP4** | 0.801 | 0.0032 | (7; 8; 18; 21; 22) |
| NM_001122659 | **EDNRB** | -0.461 | 0.0039 | (4) |
| AF026939 | **IFIT3** | 0.858 | 0.0039 | (19) |
| AK127636 | **IFNGR1** | 0.550 | 0.0032 |  |
| NM_001099857 | **IKBKG** | 0.226 | 0.0124 |  |
| X53587 | **ITGB4** | 0.608 | 0.0042 |  |
| AK090554 | **KIRREL** | 0.658 | 0.0036 |  |
| NM_177457 | **LYNX1** | 0.716 | 0.0033 |  |
| BC071579 | **MAP4K3** | -0.387 | 0.0043 | (27) |
| BC068497 | **MAPKAPK3** | 0.246 | 0.0044 |  |
| AK122767 | **MAPKAPK5** | -0.191 | 0.0057 |  |
| AB023190 | **MAST1** | -0.302 | 0.0053 |  |
| AK309963 | **MDK** | 0.829 | 0.0030 | (6; 16) |
| NM_022463 | **NXN** | 0.946 | 0.0035 |  |
| BC113557 | **OR5P2** | -0.080 | 0.0114 |  |
| NM_176871 | **PDLIM2** | 0.314 | 0.0054 |  |
| BQ931456 | **PLA2G16** | 0.467 | 0.0056 |  |
| BM911684 | **PPP1R14B** | 0.558 | 0.0037 | (4) |
| AK122881 | **PRICKLE1** | 0.940 | 0.0028 |  |
| BM544109 | **PRNPIP** | -0.253 | 0.0041 |  |
| CR749277 | **PTPRK** | 0.088 | 0.0067 |  |
| BC041333 | **RAP2A** | 0.478 | 0.0027 | (5) |
| CD388106 | **S100A10** | 0.261 | 0.0057 | (13; 26) |
| BQ683841 | **S100A11** | 0.488 | 0.0061 | (9; 17; 27) |
| NM_017789 | **SEMA4C** | 0.356 | 0.0044 |  |
| BC053671 | **SH3RF1** | -0.366 | 0.0047 |  |
| NM_003253 | **TIAM1** | 0.587 | 0.0078 | (22) |
| NM_001065 | **TNFRSF1A** | 0.666 | 0.0041 |  |
| NM_138440 | **VASN** | 0.644 | 0.0041 |  |
| NM_001025366 | **VEGFA** | -0.629 | 0.0053 | (5; 27) |
| **Cell cycle, proliferation** | |  |  |  |
| NM_024095 | **ASB8** | -0.321 | 0.0056 |  |
| AK027837 | **BZW2** | -0.537 | 0.0032 |  |
| CR600892; AB209095 | **CDC2L1; CDC2L2** | -0.263 | 0.0055 | (16) |
| AK307066 | **CKS1B** | 0.397 | 0.0067 | (27) |
| NM_080927 | **DCBLD2** | 0.261 | 0.0054 | (27) |
| NM_016033 | **FAM82B** | -0.528 | 0.0041 |  |
| NM_003913 | **PRPF4B** | -0.484 | 0.0034 | (13) |
| BC023552 | **SFN** | 0.556 | 0.0035 | (25; 17; 22) |
| U44429 | **TPD52L1** | 0.399 | 0.0045 | (13) |
| BM924710 | **TSPO** | 0.547 | 0.0025 |  |
| BC036049 | **UBE2CBP** | -1.104 | 0.0038 |  |
| NM_004724 | **ZW10** | -0.301 | 0.0058 |  |
| **Metabolism** |  |  |  |  |
| NM_001995 | **ACSL1** | -0.610 | 0.0040 |  |
| DQ345298 | **AGPAT9** | -0.318 | 0.0061 |  |
| NM_000698 | **ALOX5** | 1.240 | 0.0027 | (22) |
| NM_004044 | **ATIC** | 0.489 | 0.0046 |  |
| NM_004776 | **B4GALT5** | 0.506 | 0.0048 |  |
| NM_203314 | **BDH1** | -0.558 | 0.0032 | (7; 8) |
| BX647369 | **CSGALNACT2** | 0.539 | 0.0039 |  |
| NM_000104 | **CYP1B1** | 1.164 | 0.0032 | (7; 8; 22; 25) |
| NM_000403 | **GALE** | 1.012 | 0.0041 | (9; 12; 27) |
| NM_001482 | **GATM** | -0.986 | 0.0049 | (9; 17) |
| NM_000187 | **HGD** | -0.916 | 0.0046 | (6; 8; 12; 13; 16; 18) |
| NM_022132 | **MCCC2** | -0.587 | 0.0040 |  |
| NM_003551 | **NME5** | -0.348 | 0.0052 |  |
| NM_000919 | **PAM** | 0.390 | 0.0038 | (13; 17) |
| AK226121 | **PCYT1B** | -0.068 | 0.0035 | (14) |
| NM_024829 | **PLBD1** | 0.428 | 0.0040 |  |
| CR609147 | **PRPSAP1** | -0.530 | 0.0039 |  |
| BF219474 | **RPL27** | 0.160 | 0.0043 |  |
| CR936821 | **SLC15A4** | 0.377 | 0.0033 |  |
| BC041945 | **SLC27A6** | 0.848 | 0.0039 | (17; 22) |
| NM_003115 | **UAP1** | 0.402 | 0.0049 | (27) |
| CR592620 | **UBE2E2** | 0.128 | 0.0052 |  |
| BC007348 | **UPP1** | 0.780 | 0.0030 | (16; 27) |
| NM_017414 | **USP18** | 0.616 | 0.0041 |  |
| **Cytoskeleton, membrane** | |  |  |  |
| NM_173465 | **COL23A1** | -0.823 | 0.0029 | (22) |
| NM_080881 | **DBN1** | 0.428 | 0.0019 |  |
| BM920638 | **DCTN3** | -0.353 | 0.0046 |  |
| NM_001935 | **DPP4** | 0.746 | 0.0036 | (2;10; 12; 15; 16; 17; 18; 22; 25; 27) |
| BC040558 | **DYNC2LI1** | -0.305 | 0.0039 |  |
| BC018798 | **FXYD5** | 1.005 | 0.0031 | (9) |
| AK125670 | **HPN** | 0.199 | 0.0072 | (7) |
| AJ406940 | **KRTAP4-8** | 0.220 | 0.0058 |  |
| AF414442 | **MUC16** | -0.230 | 0.0025 |  |
| NM_015332 | **NUDCD3** | 0.115 | 0.0047 |  |
| NM_173462 | **PAPLN** | 0.469 | 0.0035 |  |
| AL832205 | **PHLDB2** | 0.313 | 0.0047 |  |
| NM_020717 | **SHROOM4** | 0.798 | 0.0043 |  |
| BG483345 | **SLPI** | 0.667 | 0.0039 | (7; 8; 22) |
| NM_021069 | **SORBS2** | -0.774 | 0.0031 | (6; 7; 13; 22; 28) |
| AK094603 |  | -0,693 | 0.0041 |  |
| AB032961 | **SPIRE1** | 0.470 | 0.0057 |  |
| NM_014767 | **SPOCK2** | 1.158 | 0.0035 | (24) |
| NM_001079515 | **TBCE** | -0.557 | 0.0044 | (27) |
| **Intracellular trafficking** | |  |  |  |
| AF085692 | **ABCC3** | 1.154 | 0.0031 | (10; 18; 22; 27) |
| NM_005829 | **AP3S2** | -0.369 | 0.0062 |  |
| BX537729 | **BCAP29** | -0.594 | 0.0039 | (5) |
| NM_016429 | **COPZ2** | -0.346 | 0.0062 |  |
| NM_015268 | **DNAJC13** | -0.308 | 0.0048 |  |
| NM_014601 | **EHD2** | 0.453 | 0.0037 |  |
| NM_014600 | **EHD3** | -0.171 | 0.0041 |  |
| AK096403 | **EMP2** | 0.800 | 0.0035 |  |
| NM_001424 |  | 0,519 | 0.0048 |  |
| AK025330 | **GBF1** | 0.318 | 0.0044 |  |
| BC146763 | **MYO1D** | 0.518 | 0.0047 |  |
| AK128739 | **NUCB2** | -0.379 | 0.0062 | (27; 28) |
| NM_015087 | **SPG20** | -0.395 | 0.0058 |  |
| AK024381 | **SYT12** | 0.568 | 0.0022 | (22) |
| BU199072 | **VAMP8** | 0.423 | 0.0043 | (27) |
| **Transcription factors** | |  |  |  |
| NM_012097 | **ARL5A** | -0.439 | 0.0053 |  |
| NM_003203 | **C2orf3** | -0.327 | 0.0034 |  |
| NM_004143 | **CITED1** | 1.075 | 0.0047 | (6; 7; 8; 10; 11; 15; 16; 17; 18; 22) |
| NM_001110514 | **EBF4** | -0.741 | 0.0043 |  |
| BC064700 | **ESRRG** | -0.645 | 0.0042 |  |
| NM_002018 | **FLII** | 0.546 | 0.0043 | (17; 27) |
| CR594049 | **MDFI** | 0.497 | 0.0037 |  |
| NM_004529 | **MLLT3** | -0.568 | 0.0049 |  |
| AB209742 | **PARP9** | 0.535 | 0.0049 | (26) |
| NM_002655 | **PLAG1** | 0.731 | 0.0057 | (25) |
| NM_004348 | **RUNX2** | 0.705 | 0.0033 | (22) |
| NM_003107 | **SOX4** | 0.569 | 0.0028 | (7; 21; 27) |
| AL833496 | **TAF10** | 0.074 | 0.0067 |  |
| BC064698 | **TFCP2L1** | -0.357 | 0.0043 | (22) |
| CR749798 | **UBP1** | 0.198 | 0.0053 |  |
| **Stress response** |  |  |  |  |
| NM_000689 | **ALDH1A1** | -0.959 | 0.0039 | (20) |
| AB209651 | **ALDH3B1** | 0.256 | 0.0039 |  |
| NM_014674 | **EDEM1** | 0.360 | 0.0047 |  |
| BC064697 | **KIAA0247** | 0.486 | 0.0028 | (3) |
| NM_004289 | **NFE2L3** | 0.762 | 0.0028 | (21; 22) |
| BC041798 | **POLK** | -0.386 | 0.0056 |  |
| BC030786 | **PRRG1** | 0.386 | 0.0051 |  |
| NM_014614 | **PSME4** | -0.270 | 0.0052 |  |
| AK124952 | **QDPR** | -0.495 | 0.0032 |  |
| BQ226060 | **REXO2** | 0.378 | 0.0034 |  |
| NM_017827 | **SARS2** | 0.283 | 0.0059 |  |
| CR936874 | **STK25** | -0.171 | 0.0066 |  |
| NM_030755 | **TXNDC1** | -0.497 | 0.0040 | (27) |
| **Apoptosis** |  |  |  |  |
| AK092391 | **CST6** | 0.788 | 0.0039 | (6; 16; 23) |
| NM_018370 | **DRAM** | 0.879 | 0.0028 |  |
| CB993249 | **ETHE1** | 0.893 | 0.0036 | (9) |
| NM_002444 | **MSN** | 0.670 | 0.0049 | (5) |
| AF289602 | **PLSCR3** | 0.378 | 0.0021 |  |
| NM_016373 | **WWOX** | -0.484 | 0.0045 | (6; 9; 17) |
| **Tumorigenesis** |  |  |  |  |
| AB209922 | **DNAJB4** | -0.488 | 0.0036 | (9) |
| BC022544 | **NAP1L5** | -0.382 | 0.0064 |  |
| BG189312 | **OCIAD2** | 0.306 | 0.0059 |  |
| BX641112 | **TUSC3** | 0.809 | 0.0042 | (10; 15; 16; 17; 22; 27) |
| AY366508 | **VWA5A** | 0.416 | 0.0053 |  |
| **Ions channel and transporter** | |  |  |  |
| AK094673 | **ATP1B3** | 0.486 | 0.0036 |  |
| AB014603 | **ATP2C2** | -0.025 | 0.0045 | (9) |
| NM_017688 | **BSPRY** | -0.509 | 0.0043 | (27) |
| NM_002250 | **KCNN4** | 0.791 | 0.0041 | (7; 8; 13; 22) |
| BM684446 | **MT1A** | -0.846 | 0.0045 | (7; 8; 9; 16; 25) |
| BC036352 | **SCNN1B** | 0.380 | 0.0044 |  |
| NM_052832 | **SLC26A7** | -1.243 | 0.0024 | (9; 22; 25) |
| NM_001098484 | **SLC4A4** | -1.075 | 0.0026 | (13; 22; 25; 27) |
| AK074065 | **TMC6** | 1.078 | 0.0035 | (9; 22) |
| AK126955 | **TTYH2** | -0.545 | 0.0053 |  |
| **Immune response, inflammation, chemokines** | | |  |  |
| BU729350 | **DEFA5** | -0.542 | 0.0042 |  |
| NM_002182 | **IL1RAP** | 0.794 | 0.0028 | (25; 27) |
| BC062422 | **IL27** | 0.926 | 0.0030 |  |
| AK023969 | **NOD1** | 0.746 | 0.0028 |  |
| CR607707 | **PLP2** | 0.867 | 0.0030 | (1) |
| CR594071 | **SERPINA1** | 1.156 | 0.0038 | (6; 7; 8; 13; 15; 16; 18; 17; 22) |
| NM_000062 | **SERPING1** | 0.989 | 0.0038 |  |
| **Mitochondria** |  |  |  |  |
| BM546299 | **RAB32** | 0.514 | 0.0041 |  |
| NM_001033568 | **RHOT1** | -1.006 | 0.0024 |  |
| NM_031947 | **SLC25A2** | -0.275 | 0.0074 |  |
| AL110179 | **SLC25A40** | -0.412 | 0.0045 |  |
| **Unknown function** | |  |  |  |
| BC098410 | **ANO6** | 0.461 | 0.0038 |  |
| AK097018 | **ARMCX6** | 0.457 | 0.0052 |  |
| NM_198075, NM_173573 | **C11orf35; LRRC56** | -0.387 | 0.0039 |  |
| AK090484 | **C17orf62** | 0.404 | 0.0042 |  |
| CR592917 | **C6orf1** | 0.310 | 0.0053 |  |
| AL834122 | **C8orf76** | -0.382 | 0.0079 |  |
| AK055796 | **CAPSL** | -0.386 | 0.0078 |  |
| NM_144647 | **CSRNP2** | 0.187 | 0.0041 |  |
| AK023916 | **DEPDC6** | -0.815 | 0.0041 | (22) |
| NM_015036 | **ENDOD1** | 0.806 | 0.0039 | (17) |
| NM_152678 | **FAM116A** | -0.600 | 0.0039 |  |
| AL832346 | **FAM126B** | -0.143 | 0.0045 |  |
| AJ298133 | **FAM167A** | -0.797 | 0.0037 | (22; 28) |
| BX648723 | **FAM179B** | -0.323 | 0.0036 |  |
| NM_001029885 | **GLTPD1** | 0.128 | 0.0046 |  |
| AK075322 | **GSG1** | -0.403 | 0.0040 |  |
| NM_017912 | **HERC6** | 0.275 | 0.0043 |  |
| NM_001034841 | **ITPRIPL2** | 0.620 | 0.0030 |  |
| BC031038 | **KCTD17** | 0.310 | 0.0045 |  |
| NM_014701 | **KIAA0256** | -0.329 | 0.0064 | (27) |
| BM923201 | **LOC284422** | -0.465 | 0.0053 |  |
| AK091057 | **LOC285535** | -0.492 | 0.0044 |  |
| AK097526 | **LOC285957** | 0.178 | 0.0059 |  |
| XM_001128367 | **LOC439949** | -0.466 | 0.0039 |  |
| BX537972 | **LYSMD3** | -0.328 | 0.0028 |  |
| BC032998 | **MANSC1** | -0.066 | 0.0088 |  |
| NM_152637 | **METTL7B** | 0.730 | 0.0045 |  |
| AK125512 | **MOSC2** | -0.757 | 0.0039 |  |
| AK125286 | **MST150** | 0.311 | 0.0058 | (24) |
| BC094756 | **MTMR11** | 0.584 | 0.0030 | (27) |
| NM_144653 | **NACC2** | -0.458 | 0.0056 |  |
| CR602296 | **NT5C3L** | -0.584 | 0.0038 |  |
| AB208908 | **PLEKHA4** | 0.914 | 0.0040 |  |
| AK092742 | **PLEKHJ1** | -0.521 | 0.0046 |  |
| NM_024776 | **SGK269** | 0.266 | 0.0055 |  |
| NM_138356 | **SHF** | 0.051 | 0.0052 |  |
| CR749477 | **SPAG16** | -0.166 | 0.0064 |  |
| BC025748 | **TBCCD1** | -0.193 | 0.0051 |  |
| AK027125 | **tcag7.903** | -0.398 | 0.0048 |  |
| CR617078 | **TMEM98** | 0.091 | 0.0022 |  |
| NM_018639 | **WSB2** | -0.509 | 0.0035 |  |
| BX647283 | **WSCD2** | -0.609 | 0.0057 |  |
| U82319 | **YDD19** | -0.457 | 0.0047 |  |
| AK023904 | **ZMAT4** | -0.713 | 0.0060 | (9; 25) |
| AK092450 |  | 0.457 | 0.0038 |  |
| AL049227 |  | 0.639 | 0.0040 |  |
| BC030766 |  | 0.292 | 0.0045 |  |
| BC035116 |  | -0.739 | 0.0045 |  |
| BC012900 |  | 0.704 | 0.0035 |  |
| AK123110 |  | -0.042 | 0.0039 |  |
| AK055000 |  | -0.590 | 0.0041 |  |
| BX093146 |  | -0.249 | 0.0042 |  |
| XM_353094 |  | -0.636 | 0.0045 |  |
| XM_353427 |  | -0.348 | 0.0064 |  |
| XM_172855 |  | 0.634 | 0.0049 |  |
| XM_353045 |  | 0.212 | 0.0060 |  |
| XM_351314 |  | 0.369 | 0.0035 |  |
| NM_032710 |  | -0.146 | 0.0050 |  |
| XM_353503 |  | -0.166 | 0.0036 |  |
| XM_294028 |  | -0.077 | 0.0044 |  |
| AL137446 |  | -0.499 | 0.0046 |  |
| NM_053040 |  | 0.562 | 0.0038 |  |

**1**: Barden CB, Clin Cancer Res, 9:1792-800, 2003; **2**: Borrello MG, Proc Natl Acad Sci U S A, 102:14825-30, 2005; **3**: Cahill S, Mol Cancer, 5:70, 2006; **4**: Cerutti JM, J Clin Invest, 113:1234-42, 2004; **5**: Chevillard S, Clin Cancer Res, 10:6586-97, 2004; **6**: Durand S, J Clin Endocrinol Metab, 93:1195-202, 2008; **7**: Finley DJ, J Clin Endocrinol Metab, 89:3214-23, 2004;.**8**: Finley DJ, Ann Surg, 240:425-36, 2004; **9**: Finn SP, Virchows Arch, 450:249-60, 2007; **10**: Fluge O, Thyroid, 16:161-75, 2006; **11**: Fryknas M, Tumour Biol, 27:211-20, 2006; **12**: Fujarewicz K, Endocr Relat Cancer, 14:809-26, 2007; **13**: Giordano TJ, Oncogene, 24:6646-56, 2005; **14**: Gombos K, Cancer Genomics Proteomics, 4:403-10, 2007; **15**: Griffith OL, J Clin Oncol, 24:5043-51, 2006; **16**: Huang Y, Proc Natl Acad Sci U S A, 98:15044-9, 2001; **17**: Jarzab B, Cancer Res, 65:1587-97, 2005; **18**: Lubitz CC, J Mol Diagn, 8:490-8, 2006; **19**: Mesa C, Cancer Res, 66:6521-9, 2006; **20**: Montero-Conde C, Oncogene, 27:1554-61, 2008; **21**: Murphy KM, Hum Pathol, 39:420-6, 2008; **22**: Nikolova DN, Oncol Rep, 20:105-21, 2008; **23**: Oler G, Clin Cancer Res, 14:4735-42, 2008; **24**: Port M, Radiat Res, 168:639-49, 2007; **25**: Prasad NB, Clin Cancer Res, 14:3327-37, 2008; **26**: Puxeddu E, Endocr Relat Cancer, 12:319-34, 2005; **27**: Salvatore G, Cancer Res, 67:10148-58, 2007; **28**: Wreesmann VB, Cancer Res, 64:3780-9, 2004.
