# Supplementary material for: Strategy to Find Molecular Signatures in a Small Series of Rare Cancers: Validation for Radiation-Induced Breast and Thyroid Tumors
Source: PLoS One. 2011 Aug 11;6(8):e23581. doi: 10.1371/journal.pone.0023581 (PMC3154936; doi:10.1371/journal.pone.0023581)
Supplement: Table S2 — RMS values. Testing tumors were classified by considering the RMS in the respective validation spaces (see Material and Methods: Aid to classification of testing tumors). The delta RMS threshold is the shortest distance between the two RMS scatter plots of the learning tumor minus the sum of the standard deviation to the barycenter of each RMA scatter plot. A: Thyroid tumors series (FTA vs PTC). B: Post-Chernobyl series. C: Post-radiotherapy breast cancer series. FTA: follicular thyroid adenoma, PTC: papillary thyroid carcinoma, R: radiation-induced, S: sporadic, ?: unclassified. (DOC) [file pone.0023581.s005.doc]

**RMS values**

**A**

| **Tumors** | **Histology** | **RMS_FTA_^class^** | **Prediction** | **RMS_PTC_^class^** | **ΔRMS**  Limite delta 0.02 |
| --- | --- | --- | --- | --- | --- |
| X1 | FTA | 0.303014 | FTA | 0.415028 | 0.112014 |
| X2 | FTA | 0.189515 | FTA | 0.477365 | 0.287850 |
| X3 | FTA | 0.337842 | FTA | 0.396941 | 0.059099 |
| X4 | FTA | 0.247291 | FTA | 0.457985 | 0.210694 |
| X5 | FTA | 0.302806 | FTA | 0.421715 | 0.118910 |
| X6 | FTA | 0.197231 | FTA | 0.474871 | 0.277640 |
| X7 | FTA | 0.314091 | FTA | 0.406283 | 0.092193 |
| X8 | FTA | 0.332584 | FTA | 0.403644 | 0.071060 |
| X9 | FTA | 0.246423 | FTA | 0.451369 | 0.204946 |
| X10 | FTA | 0.215324 | FTA | 0.461703 | 0.246379 |
| X11 | FTA | 0.280178 | FTA | 0.441478 | 0.161300 |
| X12 | FTA | 0.248614 | FTA | 0.450969 | 0.202355 |
| X14 | FTA | 0.257353 | FTA | 0.448914 | 0.191561 |
| X15 | FTA | 0.248905 | FTA | 0.446013 | 0.197108 |
| X16 | FTA | 0.650376 | FTA | 0.899999 | 0.249623 |
| X17 | PTC | 0.507046 | PTC | 0.215141 | 0.291905 |
| X18 | PTC | 0.527504 | PTC | 0.182545 | 0.344960 |
| X19 | PTC | 0.440429 | PTC | 0.303547 | 0.136882 |
| X20 | PTC | 0.465101 | PTC | 0.283883 | 0.241951 |
| X21 | PTC | 0.425886 | PTC | 0.321339 | 0.104547 |
| X22 | PTC | 0.494521 | PTC | 0.228896 | 0.265624 |
| X23 | PTC | 0.515274 | PTC | 0.209236 | 0.306038 |
| X24 | PTC | 0.523315 | PTC | 0.192680 | 0.330634 |
| X25 | PTC | 0.473430 | PTC | 0.258016 | 0.215414 |
| X26 | PTC | 0.443063 | PTC | 0.294970 | 0.148093 |
| X27 | PTC | 0.469478 | PTC | 0.260259 | 0.209220 |
| X28 | PTC | 0.525264 | PTC | 0.164635 | 0.360629 |
| X29 | PTC | 0.415573 | PTC | 0.328227 | 0.087347 |

**B**

| **Tumors** | **Etiology** | **RMS_S_^class^** | **Prediction** | **RMS_R_^class^** | **ΔRMS**  Limite delta 0.015 |
| --- | --- | --- | --- | --- | --- |
| PTC18 | S | 0.408044 | ? | 0.400582 | 0.007462 |
| PTC19 | S | 0.385920 | S | 0.431181 | 0.045261 |
| PTC21 | S | 0.362152 | S | 0.467718 | 0.105565 |
| PTC22 | S | 0.295438 | S | 0.482808 | 0.187370 |
| PTC25 | S | 0.302401 | S | 0.504750 | 0.202350 |
| PTC20 | S | 0.327827 | S | 0.500070 | 0.172242 |
| PTC26 | S | 0.376022 | S | 0.423272 | 0.047249 |
| S404 | R | 0.439619 | R | 0.365189 | 0.074430 |
| S405 | R | 0.438858 | R | 0.351583 | 0.087275 |
| S423 | R | 0.385066 | R | 0.400502 | 0.015436 |
| S425 | R | 0.457996 | R | 0.340567 | 0.117429 |
| V519 | R | 0.432076 | R | 0.396958 | 0.035117 |
| V608 | R | 0.435516 | R | 0.400186 | 0.035330 |

**C**

| **Tumors** | **Etiology** | **RMS_S_^class^** | **Prediction** | **RMS_R_^class^** | **ΔRMS**  Limite delta 0.02 |
| --- | --- | --- | --- | --- | --- |
| BfHL8 | R | 0.590661 | R | 0.333262 | 0.257399 |
| BfHL31 | R | 0.476337 | ? | 0.470118 | 0.006219 |
| BfHL43 | R | 0.537165 | R | 0.362688 | 0.174477 |
| BfHL48 | R | 0.543757 | R | 0.405206 | 0.138551 |
| BfHL50 | R | 0.638740 | R | 0.249792 | 0.388949 |
| BfHL52 | R | 0.633405 | R | 0.252904 | 0.380500 |
| BfHL53 | R | 0.583390 | R | 0.316008 | 0.267381 |
| BfHL58 | R | 0.514733 | R | 0.429813 | 0.084920 |
| BfHL60 | R | 0.517545 | R | 0.424742 | 0.092803 |
| BfHL62 | R | 0.505732 | R | 0.446137 | 0.059595 |
| BfHL64 | R | 0.628192 | R | 0.332287 | 0.295905 |
| BfHLX1 | R | 0.548092 | R | 0.391041 | 0.157051 |
| Control5 | S | 0.393804 | S | 0.545682 | 0.151878 |
| Control7 | S | 0.319918 | S | 0.592932 | 0.273014 |
| Control10 | S | 0.493089 | ? | 0.481806 | 0.011283 |
| Control14 | S | 0.370458 | S | 0.558642 | 0.188185 |
| Control15 | S | 0.448263 | S | 0.516506 | 0.068243 |
| Control17 | S | 0.434435 | S | 0.480709 | 0.046274 |
| Control18 | S | 0.473007 | S | 0.508420 | 0.035413 |
| Control21 | S | 0.336539 | S | 0.559996 | 0.223457 |
| Control22 | S | 0.325817 | S | 0.617047 | 0.291230 |
| Control27 | S | 0.439218 | S | 0.507753 | 0.068535 |
