# Supplementary material for: Strategy to Find Molecular Signatures in a Small Series of Rare Cancers: Validation for Radiation-Induced Breast and Thyroid Tumors
Source: PLoS One. 2011 Aug 11;6(8):e23581. doi: 10.1371/journal.pone.0023581 (PMC3154936; doi:10.1371/journal.pone.0023581)
Supplement: Table S3 — List of genes (final stable signature) discriminating sporadic thyroid papillary carcinomas from post-Chernobyl thyroid papillary carcinomas. The signature was determined from a published dataset retrieved from the GEO database (http://www.ncbi.nlm.nih.gov/geo, accession number GSE3950). Differential gene expression values were calculated in the validation space as the average of log (post-Chernobyl PTC gene expression) minus the average of log (sporadic PTC gene expression), with the corresponding p value. (DOC) [file pone.0023581.s006.doc]

**List of genes (final stable signature) discriminating sporadic thyroid papillary carcinomas from post-Chernobyl thyroid papillary carcinomas**

| **Acc** | **Symbol** | **Name** | **Expression value** | **p** |
| --- | --- | --- | --- | --- |
| AF132794 | **ANAPC10** | Anaphase promoting complex subunit 10 | -0,034 | 0,0047 |
| AF230330 | **ANGPTL6** | Angiopoietin-like 6 | 0,453 | 0,0087 |
| AI494375 | **APBA3** | Amyloid beta precursor protein-binding, family A, member 3 | -0,325 | 0,0059 |
| BC012321 | **ARC** | Activity-regulated cytoskeleton-associated protein | 0,283 | 0,0092 |
| BC007848 | **ARHGAP19** | Rho GTPase activating protein 19 | 0,794 | 0,0034 |
| BF976287 | **ARPC1B** | Actin related protein 2/3 complex, subunit 1B, 41kDa | 0,397 | 0,0073 |
| AK026556 | **ATP5E** | ATP synthase, mitochondrial F1 complex, epsilon subunit | -0,093 | 0,0054 |
| AF087135 | **ATP5H** | ATP synthase, mitochondrial F0 complex, subunit d | -0,097 | 0,0088 |
| D87462 | **BAP1** | BRCA1 associated protein-1 | -0,364 | 0,0046 |
| BF338113 | **C4A** | Complement component 4A | 0,570 | 0,0069 |
| M59815 |  |  | 0,510 | 0,0071 |
| BI197164 | **CADM1** | Cell adhesion molecule 1 | 0,269 | 0,0090 |
| M94345 | **CAPG** | Capping protein (actin filament), gelsolin-like | 0,647 | 0,0044 |
| AL542005 | **CD164** | CD164 molecule, sialomucin | -0,131 | 0,0124 |
| AL558308 | **CD3E** | CD3e molecule, epsilon | 0,744 | 0,0048 |
| AI564271 | **CD97** | CD97 molecule | 0,418 | 0,0084 |
| AI206764 | **CHKB** | Choline kinase beta | 0,435 | 0,0059 |
| X14723 | **CLU** | Clusterin | 0,491 | 0,0073 |
| L00974 |  |  | 0,689 | 0,0046 |
| AL046845 | **CNN1** | Calponin 1, basic, smooth muscle | 0,532 | 0,0060 |
| BC007425 | **CSTF1** | Cleavage stimulation factor, 3' pre-RNA, subunit 1, 50kDa | -0,090 | 0,0079 |
| U15782 | **CSTF3** | Cleavage stimulation factor, 3' pre-RNA, subunit 3, 77kDa | 0,042 | 0,0060 |
| AF064087 | **CUL3** | Cullin 3 | 0,057 | 0,0041 |
| BG740758 | **CYB5A** | Cytochrome b5 type A (microsomal) | -0,027 | 0,0065 |
| D29643 | **DDOST** | Dolichyl-diphosphooligosaccharide-protein glycosyltransferase | -0,398 | 0,0034 |
| BC001121 | **DGUOK** | Deoxyguanosine kinase | -0,063 | 0,0056 |
| BG762692 | **DHCR24** | 24-dehydrocholesterol reductase | 0,439 | 0,0039 |
| AL050378 | **DKFZP586I1420** | Hypothetical protein DKFZp586I1420 | 0,228 | 0,0076 |
| AB058718 | **ERMP1** | Endoplasmic reticulum metallopeptidase 1 | 0,139 | 0,0114 |
| BE894754 | **ETF1** | Eukaryotic translation termination factor 1 | 0,096 | 0,0109 |
| BC010004 | **FDPS** | Farnesyl diphosphate synthase | 0,404 | 0,0070 |
| BF971255 | **FKBP1A** | FK506 binding protein 1A, 12kDa | -0,135 | 0,0045 |
| AK022628 | **FOXK1** | Forkhead box K1 | -0,194 | 0,0052 |
| U58975 | **FRAT1** | Frequently rearranged in advanced T-cell lymphomas | 0,099 | 0,0070 |
| BF346549 | **GPR17** | G protein-coupled receptor 17 | 0,129 | 0,0076 |
| AB033046 | **GRID1** | Glutamate receptor, ionotropic, delta 1 | -0,046 | 0,0104 |
| Z30094 | **GTF2H2** | General transcription factor IIH, polypeptide 2, 44kDa | 0,316 | 0,0066 |
| L24758 | **IBSP** | Integrin-binding sialoprotein | 0,064 | 0,0075 |
| V00534 | **IFNB1** | Interferon, beta 1, fibroblast | -0,294 | 0,0048 |
| X04434 | **IGF1R** | Insulin-like growth factor 1 receptor | 0,120 | 0,0069 |
| BC005007 | **IGFBP6** | Insulin-like growth factor binding protein 6 | 0,457 | 0,0047 |
| AL117607 | **LOC203274** | Hypothetical protein LOC203274 | -0,011 | 0,0097 |
| D86960 | **LPGAT1** | Lysophosphatidylglycerol acyltransferase 1 | 0,251 | 0,0127 |
| BC006143 | **MAFG** | V-maf oncogene homolog G | -0,586 | 0,0056 |
| BI259434 | **MAP3K14** | Mitogen-activated protein kinase kinase kinase 14 | -0,220 | 0,0103 |
| AF321561 | **MASP2** | Mannan-binding lectin serine peptidase 2 | 0,001 | 0,0100 |
| Z48051 | **MOG** | Myelin oligodendrocyte glycoprotein | -0,332 | 0,0035 |
| L34600 | **MTIF2** | Mitochondrial translational initiation factor 2 | 0,259 | 0,0064 |
| AL135262 | **MYO1C** | Myosin IC | 0,210 | 0,0078 |
| BC012151 | **NFX1** | Nuclear transcription factor, X-box binding 1 | 0,010 | 0,0107 |
| AJ002535 | **OBSCN** | Obscurin | -0,043 | 0,0078 |
| Y00345 | **PABPC1** | Poly(A) binding protein, cytoplasmic 1 | -0,144 | 0,0076 |
| M37721 | **PAM** | Peptidylglycine alpha-amidating monooxygenase | 0,635 | 0,0049 |
| L12686 | **PDE4B** | Phosphodiesterase 4B, cAMP-specific | -1,003 | 0,0035 |
| U82328 | **PDHX** | Pyruvate dehydrogenase complex, component X | 0,198 | 0,0089 |
| AK025729 | **PELO** | Pelota homolog | -0,216 | 0,0032 |
| X52638 | **PFKFB1** | 6-phosphofructo-2-kinase/fructose-2,6-biphosphatase 1 | 0,380 | 0,0053 |
| BC007952 | **PKM2** | Pyruvate kinase, muscle | 0,091 | 0,0086 |
| M98252 | **PLOD1** | Procollagen-lysine 1, 2-oxoglutarate 5-dioxygenase 1 | -0,405 | 0,0034 |
| M27602 | **PRSS1** | Protease, serine, 1 | 0,654 | 0,0069 |
| X71345 | **PRSS3** | Protease, serine, 3 | 0,741 | 0,0044 |
| BC008414 | **PSMB7** | Proteasome subunit, beta type, 7 | 0,006 | 0,0079 |
| BE784260 | **PSME4** | Proteasome activator subunit 4 | -0,359 | 0,0085 |
| M24406 | **PVR** | Poliovirus receptor | -0,167 | 0,0076 |
| U84138 | **RAD51L1** | RAD51-like 1 | 0,471 | 0,0067 |
| X08004 | **RAP1B** | RAP1B, member of RAS oncogene family | -0,339 | 0,0098 |
| BC003403 | **RAP2C** | RAP2C, member of RAS oncogene family | 0,140 | 0,0099 |
| AU143092 | **RBM3** | RNA binding motif protein 3 | 0,052 | 0,0112 |
| AL136924 | **RIN2** | Ras and Rab interactor 2 | 0,350 | 0,0083 |
| AL049999 | **RSL1D1** | Ribosomal L1 domain containing 1 | -0,055 | 0,0047 |
| AJ290445 | **SARM1** | Sterile alpha and TIR motif containing 1 | -0,201 | 0,0047 |
| Z92980 | **SCNN1A** | Sodium channel, nonvoltage-gated 1 alpha | 0,330 | 0,0108 |
| BG257925 | **SEC61B** | Sec61 beta subunit | -0,348 | 0,0045 |
| L40377 | **SERPINB8** | Serpin peptidase inhibitor, clade B, member 8 | 0,157 | 0,0107 |
| BG829624 | **SERPINE1** | Serpin peptidase inhibitor, clade E, member 1 | -0,491 | 0,0060 |
| AI249743 | **SERPINF2** | Serpin peptidase inhibitor, clade F, member 2 | -0,186 | 0,0068 |
| AV755996 | **SHFM1** | Split hand/foot malformation type 1 | -0,037 | 0,0039 |
| BG762583 | **SIRPA** | Signal-regulatory protein alpha | 0,372 | 0,0071 |
| AF226053 | **SMYD2** | SET and MYND domain containing 2 | 0,204 | 0,0044 |
| W46548 |  |  | 0,199 | 0,0084 |
| BE266406 | **SRCAP** | Snf2-related CREBBP activator protein | 0,224 | 0,0031 |
| AF110647 | **SSR3** | Signal sequence receptor, gamma | -0,069 | 0,0081 |
| U52426 | **STIM1** | Stromal interaction molecule 1 | -0,710 | 0,0048 |
| BC007835 | **STK40** | Serine/threonine kinase 40 | -0,610 | 0,0030 |
| BF966739 | **SUMO3** | SMT3 suppressor of mif two 3 homolog 3 | 0,115 | 0,0106 |
| L34587 | **TCEB1** | Transcription elongation factor B (SIII), polypeptide 1 | -0,117 | 0,0057 |
| AF017789 | **TCERG1** | Transcription elongation regulator 1 | -0,229 | 0,0069 |
| L10386 | **TGM3** | Transglutaminase 3 | 0,321 | 0,0074 |
| AB055251 | **THNSL1** | Threonine synthase-like 1 | 0,416 | 0,0065 |
| AF183409 | **TMEM165** | Transmembrane protein 165 | -0,192 | 0,0050 |
| CAC39753 | **TMEM178** | Transmembrane protein 178 | 0,238 | 0,0114 |
| BC011369 | **TRAPPC2L** | Trafficking protein particle complex 2-like | 0,040 | 0,0083 |
| M63582 | **TRH** | Thyrotropin-releasing hormone | -0,099 | 0,0036 |
| AF230393 | **TRIM27** | Tripartite motif-containing 27 | 0,192 | 0,0084 |
| BG913323 | **VCP** | Valosin-containing protein | 0,227 | 0,0092 |
| BC001440 | **YWHAE** | 14-3-3epsilon | 0,084 | 0,0069 |
| AK024049 | **ZCCHC17** | Zinc finger, CCHC domain containing 17 | -0,062 | 0,0094 |
| AB046779 | **ZFP14** | Zinc finger protein 14 homolog | 0,236 | 0,0090 |
| AAH06118 | **ZKSCAN3** | Zinc finger with KRAB and SCAN domains 3 | 0,259 | 0,0056 |
| AK024442 | **ZNF160** | Zinc finger protein 160 | 0,114 | 0,0075 |
| AK027834 | **ZNF585B** | Zinc finger protein 585B | 0,060 | 0,0032 |
| AK022668 | **ZNF669** | Zinc finger protein 669 | 0,208 | 0,0046 |
| V00590 | **RNU1A** | Non-coding RNA | -0,220 | 0,0089 |
| AF019413 | Not in Hs cluster | Homo sapiens HLA class III region | 0,670 | 0,0067 |
| AL163202 | Not in Hs cluster | Homo sapiens chromosome 21 segment HS21C002 | 0,337 | 0,0048 |
| AP000356 | Not in Hs cluster | Homo sapiens genomic DNA, chromosome 22q11.2 | 0,261 | 0,0053 |
| AP001576 | Not in Hs cluster | Homo sapiens genomic DNA, chromosome 6q25.2 | 0,212 | 0,0066 |
| AP001677 | Not in Hs cluster | Homo sapiens genomic DNA, chromosome 21q | 0,267 | 0,0078 |
| L43338 | Not in Hs cluster | Homo sapiens chromosome 2 | -0,227 | 0,0064 |
