# Supplementary material for: Strategy to Find Molecular Signatures in a Small Series of Rare Cancers: Validation for Radiation-Induced Breast and Thyroid Tumors
Source: PLoS One. 2011 Aug 11;6(8):e23581. doi: 10.1371/journal.pone.0023581 (PMC3154936; doi:10.1371/journal.pone.0023581)
Supplement: Table S4 — List of genes (final stable signature) discriminating sporadic breast cancers from post-radiotherapy breast cancers. The signature was determined from a published dataset retrieved from the ArrayExpress database (http://www.ebi.ac.uk/microarray-as/ae, accession number E-NCMF-30). Differential gene expression values were calculated in the validation space as the average of log (control breast tumor gene expression) minus the average of log (radiation-induced breast tumor gene expression), with the corresponding p value. (DOC) [file pone.0023581.s007.doc]

**List of genes (final stable signature) discriminating sporadic breast cancers from post-radiotherapy breast cancers**

| **Acc** | **Symbol** | **Name** | **Expression value** | **p** |
| --- | --- | --- | --- | --- |
| ENST00000225941 | **ABI3** | ABI family, member 3 | 0,221 | 0,0043 |
| ENST00000272102 | **ARF1** | ADP-ribosylation factor 1 | 0,013 | 0,0049 |
| ENST00000272102 |  |  | 0,011 | 0,0049 |
| ENST00000244769 | **ATXN1** | Ataxin 1 | 0,100 | 0,0051 |
| ENST00000255498 | **BEX2** | Bbrain expressed X-linked 2 | -0,212 | 0,0063 |
| ENST00000262498 | **C16orf80** | Chromosome 16 open reading frame 80 | -0,073 | 0,0042 |
| ENST00000229583 | **C6orf105** | Chromosome 6 open reading frame 105 | -0,162 | 0,0052 |
| ENST00000295023 | **CNIH4** | Cornichon homolog 4 (Drosophila) | -0,306 | 0,0051 |
| ENST00000321208 | **COQ9** | Coenzyme Q9 homolog (S. cerevisiae) | -0,282 | 0,0048 |
| ENST00000263511 | **CROCCP3** | Ciliary rootlet coiled-coil, rootletin pseudogene 3 | 0,149 | 0,0051 |
| ENST00000323761 | **DDX55** | DEAD (Asp-Glu-Ala-Asp) box polypeptide 55 | -0,215 | 0,0045 |
| ENST00000324371 | **EDC4** | Enhancer of mRNA decapping 4 | -0,167 | 0,0049 |
| ENST00000309137 | **FAM192A** | Family with sequence similarity 192, member A | -0,125 | 0,0055 |
| ENST00000326521 | **FAM76A** | Family with sequence similarity 76, member A | 0,386 | 0,0033 |
| ENST00000324505 | **FOXP2** | Forkhead box P2 | -0,492 | 0,0031 |
| ENST00000256906 | **HRH4** | Histamine receptor H4 | 0,432 | 0,0026 |
| ENST00000319496 | **LPHN2** | Latrophilin 2 | -0,181 | 0,0050 |
| ENST00000219169 | **NUTF2** | Nuclear transport factor 2 | -0,255 | 0,0054 |
| ENST00000311825 | **RIN3** | Ras and Rab interactor 3 | -0,305 | 0,0034 |
| ENST00000235150 | **RNF19B** | Ring finger protein 19B | -0,163 | 0,0044 |
| ENST00000304289 | **RP11-134K1.2** |  | -0,110 | 0,0060 |
| ENST00000222247 | **RPL18A** | Ribosomal protein L18a | -0,179 | 0,0058 |
| ENST00000221975 | **RPS19** | Ribosomal protein S19 | -0,154 | 0,0042 |
| ENST00000319461 | **RUFY1** | RUN and FYVE domain containing 1 | 0,002 | 0,0056 |
| ENST00000244529 | **SERPINB6** | Serpin peptidase inhibitor, clade B, member 6 | 0,199 | 0,0041 |
| ENST00000308755 | **SIPA1** | Signal-induced proliferation-associated 1 | 0,193 | 0,0040 |
| ENST00000268704 | **SPG7** | Spastic paraplegia 7 | -0,517 | 0,0017 |
| ENST00000295709 | **STK36** | Serine/threonine kinase 36 | 0,235 | 0,0039 |
| ENST00000255506 | **TCEAL4** | Transcription elongation factor A (SII)-like 4 | 0,109 | 0,0057 |
| ENST00000304800 | **TMEM208** | Transmembrane protein 208 | -0,061 | 0,0059 |
| ENST00000324002 | **ZC3H12A** | Zinc finger CCCH-type containing 12A | -0,153 | 0,0051 |
| ENST00000311945 | **ZMYM6** | Zinc finger, MYM-type 6 | -0,211 | 0,0053 |
| ENST00000251272 | **ZNF222** | Zinc finger protein 222 | 0,274 | 0,0036 |
| ENST00000326730 |  |  | 0,185 | 0,0043 |
| ENST00000320297 |  |  | -0,204 | 0,0048 |
| ENST00000253674 |  |  | 0,477 | 0,0035 |
| ENST00000315940 |  |  | -0,213 | 0,0028 |
| ENST00000308549 |  |  | -0,188 | 0,0056 |
| ENST00000286166 |  |  | 0,310 | 0,0042 |
| ENST00000327291 |  |  | 0,392 | 0,0045 |
| ENST00000272843 |  |  | 0,499 | 0,0033 |
| ENST00000253379 |  |  | 0,472 | 0,0026 |
| ENST00000324550 |  |  | -0,120 | 0,0033 |
| ENST00000325955 |  |  | 0,103 | 0,0036 |
| ENST00000314202 |  |  | 0,262 | 0,0046 |
