# Supplementary material for: Strategy to Find Molecular Signatures in a Small Series of Rare Cancers: Validation for Radiation-Induced Breast and Thyroid Tumors
Source: PLoS One. 2011 Aug 11;6(8):e23581. doi: 10.1371/journal.pone.0023581 (PMC3154936; doi:10.1371/journal.pone.0023581)
Supplement: Table S5 — Results of clustering obtained for the three series of tumors with the different methods. Data are given in terms of either the classification of learning tumors alone or all tumors (learning and testing). Yes: indicates that the learning set/all tumors were well clustered into two groups. No: indicates that the learning set/all tumors were not well clustered into two groups. FTA: follicular thyroid adenoma; PTC: papillary thyroid carcinoma, R: radiation-induced tumors; S: sporadic tumors; n (number of gene pairs) = 30, 55 and 860 for FTA/PTC, breast tumors and post-Chernobyl series, respectively. *Only one error. (DOC) [file pone.0023581.s008.doc]

**Results of clustering obtained for the three series of tumors with the different methods.**

| **Clustering** | **PCA** | **Bar code** | **TSP**  **1^st^ top pair** | **TSPs**  **n top pairs** | **EMts_2PCA** |
| --- | --- | --- | --- | --- | --- |
| **Learning :**  **13 FTAs vs 13 PTCs** | Yes | Yes | Yes | Yes | Yes |
| **Learning + Testing :**  **54 FTAs and PTCs** | Yes | No | No | Yes* | Yes |
| **Learning :**  **10 R vs 10 S breast tumors** | Yes | No | Yes | Yes | Yes |
| **Learning + Testing :**  **42 R and S breast tumors** | Yes | No | No | Yes | Yes |
| **learning :**  **6 R vs 7 S PTC** | Yes | No | Yes | Yes | Yes |
| **Learning + Testing :**  **26 R and S PTCs** | Yes | No | No | No | Yes |
