# Supplementary material for: Strategy to Find Molecular Signatures in a Small Series of Rare Cancers: Validation for Radiation-Induced Breast and Thyroid Tumors
Source: PLoS One. 2011 Aug 11;6(8):e23581. doi: 10.1371/journal.pone.0023581 (PMC3154936; doi:10.1371/journal.pone.0023581)
Supplement: Data S1 — Performance of the classifier. (DOC) [file pone.0023581.s009.doc]

**Performance of the classifier**

**A) Sensitivity and specificity**

Sensitivity and specificity are statistical scores of the performance of a binary-based test (the test takes two values), for example:

| ***Test values*** | **A** | **B** |
| --- | --- | --- |
| ***Test(+) = a*** | True(a) =A | False(a)=B |
| ***Test(-) = b*** | False(b)=A | True(b)=B |

***Sensitivity*** *= True(a)/(True(a)+False(b))*

***Specificity*** *= True(b)/(True(b)+False(a)).*

When the test takes three values (Test(+) ,Test(-) or Test(?), as in the EMts_2PCA method:

| ***Test values*** | **A** | **B** |
| --- | --- | --- |
| ***Test(+) = a*** | True(a)=A | False(a) =B |
| ***Test(- )= b*** | False(b) =A | True(b)=B |
| ***Test(?) = ?*** | ?=A | ?=B |

The performance of the classification can be evaluated by specificity and specificity by making a dichotomous assumption, if one of the groups is taken as reference. For example, if group A is taken as reference, the test becomes (A) versus (none-A), where the A condition is the test(+) and the none-A condition (test(none)) is the fusion of the [test(-) and test(?)].

Then ***Sensitivity*** can be defined as*: True(a) /(True(a)+[False(b)+?_(A)_])*

and ***Specificity*** as*: [True(b)+?_(B)_]/(False(a)+[ True(b)+?_(B)_]).*

For the three analyzed series of tumors, we obtained the following results depending on which group is taken as the positive reference:

| ***PTC/FTA*** | **Test(+)=(PTC)**  **Test(none)=(FTA and ?)** | **Test(+)=(FTA)**  **Test(none)=(PTC and ?)** |
| --- | --- | --- |
| ***Sensitivity*** | 1 | 1 |
| ***Specificity*** | 1 | 1 |
| **R Breast */* S Breast** | **Test(+)=(R Breast)**  **Test(none) = (S Breast and ?)** | **Test(+)=S Breast**  **Test(none)=(R Breast and ?)** |
| ***Sensitivity*** | 0.92 | 0.9 |
| ***Specificity*** | 1 | 1 |
| ***R PTC/ S PTC*** | **Test(+)=(R PTC)**  **Test(none)=(S PTC and ?)** | **Test(+)=(S PTC)**  **Test(none) = (R PTC and ?)** |
| ***Sensitivity*** | 1 | 0,86 |
| ***Specificity*** | 1 | 1 |

It therefore appears that, depending on the choice of the group taken as a positive reference, the sensitivity and specificity are not symmetrical.

**B) Relative prediction efficiency and general prediction efficiency**

In any case, the sensitivity and specificity are suitable scores for estimating the efficiency of prediction methods regarding a given group, but they do not reflect the overall efficiency of the classifier whatever the groups. Thus two new evaluators are proposed:

The relative prediction efficiency (RPE) defined as:

***RPE = (True(a) + True(b))/(True(b)+False(b)+True(b)+False(a))***

and the general prediction efficiency (GPE) defined as:

***GPE = (True(a) + True-(b))/(True(b)+False(b)+True(b)+False(a)+ ?)***

Whatever the groups, these two evaluators indicate respectively the reliability of the prediction method and its effectiveness in providing a prediction.

For the three series of samples analyzed with EMts_PCA, RPE and GPE scores are presented in Table 1.
