# Supplementary material for: Strategy to Find Molecular Signatures in a Small Series of Rare Cancers: Validation for Radiation-Induced Breast and Thyroid Tumors
Source: PLoS One. 2011 Aug 11;6(8):e23581. doi: 10.1371/journal.pone.0023581 (PMC3154936; doi:10.1371/journal.pone.0023581)
Supplement: Data S2 — Analysis of the signature discriminating thyroid follicular adenomas from thyroid papillary carcinomas. (DOC) [file pone.0023581.s010.doc]

**Analysis of the signature discriminating thyroid follicular adenomas from thyroid papillary carcinomas**

To validate the biological relevance of the signature obtained with our method of transcriptomic analysis and classification, we first checked its overlap with 50 published thyroid tumor transcriptomic analyses. Among the genes that have been shown, in these signatures, to differentiate histological subtypes of thyroid tumors and/or to be associated with the expression of thyroid oncogenes (RET/PTC isoforms or BRAFV600E), 78 genes overlapped with the present signature. Notably, 39 genes in this overlap have already been identified in signatures discriminating malignant thyroid tumors (PTC, follicular variant of PTC and/or follicular carcinomas), from benign thyroid tumors (FTA, hyperplastic nodules). Of these genes, CITED1, TUSC3, DPP4, GALE, HGD and SERPINA1 emerged as strong PTC markers in meta-analyses of previously published thyroid tumor signatures (Fujarewicz et al., 2007; Griffith et al., 2006). Our signature also included 16 over-expressed (ALOX5, DCBLD2, ENDOD1, FLII, MTMR11, NFE2L3, PAM, RUNX2, S100A10, SLC27A6, SYT12, TIAM1, TPD52L1, UAP1, UPP1 and VAMP8) and 14 under-expressed genes (BSPRY, CDC2L1, CD36, COL23A1, DEPDC6, FAM167A, KIAA0256, MAP4K3, NUCB2, PCYT1B, PRPF4B, TFCP2L1, TBCE and TXNDC1) in PTCs, which were already found to discriminate PTCs from normal thyroid tissues, suggesting that these genes could be involved in thyroid tumorigenesis. Interestingly, we found that VWA5A, a gene located at 11q23, was over-expressed in PTCs vs FTAs. Up to now, this gene has not been reported as a marker to distinguish FTAs from PTCs but, in line with our data, a loss of heterozygosity of this chromosome region was detected in 45% of FTAs and never in PTCs (Oriola et al., 2001).

Many differentially expressed genes affect signal transduction pathways, such as Wnt/βcatenin and Egf pathways, known to be essential for thyroid tumorigenesis (Aasland et al., 1990; Abbosh & Nephew, 2005; Castellone et al., 2009; Hoelting et al., 1994). These pathways promote tumorigenesis by increasing cell proliferation and regulating cell differentiation. Some genes may directly impact on Wnt/βcatenin signaling, by inhibiting the Wnt pathway through binding to disheveled proteins (PRICKLE1, NXN) or by modifying β-catenin’s availability or transactivation capacity (PTPRK, TIAM1, MDF1 and FLII). The Wnt and Egf pathways may also be regulated upstream by transcription factors such as C2orf3 and SOX4 and PLAG1 (Declercq et al., 2008; Kitadai et al., 1993; Scharer et al., 2009).

Thyroid hormone metabolism generates a high level of H_2_O_2_, which leads to permanent oxidative stress that is highly controlled by thyrocytes to avoid deleterious effects such as DNA damage, lipid peroxidation and general toxicity (Maier, van Steeg et al. 2006; Song, Driessens et al. 2007). Sporadic PTCs have a transcriptome pattern close to that of the specific B lymphocyte response against H_2_O_2_ (Detours, Delys et al. 2007). In this respect, our signature includes genes involved in the detoxification of compounds produced by lipid peroxidation (ALDH1A1, ALDH3B1), genes regulated by oxidative stress, such as transcription cofactor NFE2L3. ATP-binding cassette transporter ABCC3, serine/threonine kinase 25 (STK25), and genes coding for proteins participating in the DNA damage response, such as ETHE1, SFN, KIAA0247 and EDNRB, are also deregulated. Solute carrier family 15, member 4 (SLC15A4) displays a high affinity for carnosine, a scavenger for reactive oxygen species and for alpha-beta unsaturated aldehydes formed from peroxidation of cell membrane fatty acids during oxidative stress. It should be noted that NXN, previously mentioned in the discussion, inhibits the redox-dependent activation of the Wnt/β-catenin pathway (Funato, Michiue et al. 2006). In addition, deregulation of several genes involved in Cu and Zn ion metabolism (MTA1, TMC6) may be of importance in thyroid tumorigenesis since these ions could be antioxidants and could reduce oxidative stress in experimental hypothyroidism (Alturfan, Zengin et al. 2007), and, moreover, plasma Zn concentration decreases in patients with thyroid cancer (Al-Sayer, Mathew et al. 2004).

Aasland, R., Akslen, L.A., Varhaug, J.E. and Lillehaug, J.R. (1990) Co-expression of the genes encoding transforming growth factor-alpha and its receptor in papillary carcinomas of the thyroid. Int J Cancer, 46, 382-387.

Abbosh, P.H. and Nephew, K.P. (2005) Multiple signaling pathways converge on beta-catenin in thyroid cancer. Thyroid, 15, 551-561.

Al-Sayer, H., Mathew, T.C., Asfar, S., Khourshed, M., Al-Bader, A., Behbehani, A. and Dashti, H. (2004) Serum changes in trace elements during thyroid cancers. Mol Cell Biochem, 260, 1-5.

Alturfan, A.A., Zengin, E., Dariyerli, N., Alturfan, E.E., Gumustas, M.K., Aytac, E., Aslan, M., Balkis, N., Aksu, A., Yigit, G. et al. (2007) Investigation of zinc and copper levels in methimazole-induced hypothyroidism: relation with the oxidant-antioxidant status. Folia Biol (Praha), 53, 183-188.

Castellone, M.D., De Falco, V., Rao, D.M., Bellelli, R., Muthu, M., Basolo, F., Fusco, A., Gutkind, J.S. and Santoro, M. (2009) The beta-catenin axis integrates multiple signals downstream from RET/papillary thyroid carcinoma leading to cell proliferation. Cancer Res, 69, 1867-1876.

Declercq, J., Van Dyck, F., Van Damme, B. and Van de Ven, W.J. (2008) Upregulation of Igf and Wnt signalling associated genes in pleomorphic adenomas of the salivary glands in PLAG1 transgenic mice. Int J Oncol, 32, 1041-1047.

Detours, V., Delys, L., Libert, F., Weiss Solis, D., Bogdanova, T., Dumont, J.E., Franc, B., Thomas, G. and Maenhaut, C. (2007) Genome-wide gene expression profiling suggests distinct radiation susceptibilities in sporadic and post-Chernobyl papillary thyroid cancers. Br J Cancer, 97, 818-825.

Fujarewicz, K., Jarzab, M., Eszlinger, M., Krohn, K., Paschke, R., Oczko-Wojciechowska, M., Wiench, M., Kukulska, A., Jarzab, B. and Swierniak, A. (2007) A multi-gene approach to differentiate papillary thyroid carcinoma from

Griffith, O.L., Melck, A., Jones, S.J. and Wiseman, S.M. (2006) Meta-analysis and meta-review of thyroid cancer gene expression profiling studies identifies important diagnostic biomarkers. J Clin Oncol, 24, 5043-5051.

Funato, Y., Michiue, T., Asashima, M. and Miki, H. (2006) The thioredoxin-related redox-regulating protein nucleoredoxin inhibits Wnt-beta-catenin signalling through dishevelled. Nat Cell Biol, 8, 501-508.

Hoelting, T., Siperstein, A.E., Clark, O.H. and Duh, Q.Y. (1994) Epidermal growth factor enhances proliferation, migration, and invasion of follicular and papillary thyroid cancer in vitro and in vivo. J Clin Endocrinol Metab, 79, 401-408.

Kitadai, Y., Yamazaki, H., Yasui, W., Kyo, E., Yokozaki, H., Kajiyama, G., Johnson, A.C., Pastan, I. and Tahara, E. (1993) GC factor represses transcription of several growth factor/receptor genes and causes growth inhibition of human gastric carcinoma cell lines. Cell Growth Differ, 4, 291-296.

Maier, J., van Steeg, H., van Oostrom, C., Karger, S., Paschke, R. and Krohn, K. (2006) Deoxyribonucleic acid damage and spontaneous mutagenesis in the thyroid gland of rats and mice. Endocrinology, 147, 3391-3397.

Oriola, J., Halperin, I., Mallofre, C., Muntane, J., Angel, M. and Rivera-Fillat, F. (2001) Screening of selected genomic areas potentially involved in thyroid neoplasms. Eur J Cancer, 37, 2470-2474.

Scharer, C.D., McCabe, C.D., Ali-Seyed, M., Berger, M.F., Bulyk, M.L. and Moreno, C.S. (2009) Genome-wide promoter analysis of the SOX4 transcriptional network in prostate cancer cells. Cancer Res, 69, 709-717.

Song, Y., Driessens, N., Costa, M., De Deken, X., Detours, V., Corvilain, B., Maenhaut, C., Miot, F., Van Sande, J., Many, M.C. et al. (2007) Roles of hydrogen peroxide in thyroid physiology and disease. J Clin Endocrinol Metab, 92, 3764-3773.
